# GIRAE: a generalised approach for linking the total impact of invasion to species' range, abundance and per-unit effects

**DOI:** 10.1007/s10530-022-02836-0

**Published:** 2022-06-11

**Authors:** Guillaume Latombe, Jane A. Catford, Franz Essl, Bernd Lenzner, David M. Richardson, John R. U. Wilson, Melodie A. McGeoch

**Affiliations:** 1 Institute of Ecology and Evolution, The University of Edinburgh, King’s Buildings, EH9 3FL Edinburgh, UK; 2 Department of Geography, King’s College London, 30 Aldwych, London, WC2B 4BG UK; 3 School of Ecosystem and Forest Sciences, University of Melbourne, VIC 3121 Richmond, Australia; 4 Bioinvasions, Global Change, Macroecology Group, Department of Botany and Biodiversity Research, University of Vienna, Rennweg 14, 1030 Vienna, Austria; 5 Centre for Invasion Biology, Department of Botany and Zoology, Stellenbosch University, Stellenbosch, South Africa; 6 South African National Biodiversity Institute, Kirstenbosch Research Centre, Cape Town, South Africa; 7 Department of Ecology, Environment and Evolution, LaTrobe University, Melbourne, VIC 3086 Australia

**Keywords:** Abundance, Biological invasions, Impact, Invasive alien plant species, Vegetation management, Occupancy, South Africa

## Abstract

The total impact of an alien species was conceptualised as the product of its range size, local abundance and per-unit effect in a seminal paper by Parker et al. (Biol Invasions 1:3–19, 1999). However, a practical approach for estimating the three components has been lacking. Here, we generalise the impact formula and, through use of regression models, estimate the relationship between the three components of impact, an approach we term GIRAE (Generalised Impact = Range size × Abundance × per-unit Effect). We discuss how GIRAE can be applied to multiple types of impact, including environmental impacts, damage and management costs. We propose two methods for applying GIRAE. The species-specific method computes the relationship between impact, range size, abundance and per-unit effect for a given species across multiple invaded sites or regions of different sizes. The multi-species method combines data from multiple species across multiple sites or regions to calculate a per-unit effect for each species and is computed using a single regression model. The species-specific method is more accurate, but it requires a large amount of data for each species and assumes a constant per-unit effect for a species across the invaded area. The multi-species method is more easily applicable and data-parsimonious, but assumes the same relationship between impact, range size and abundance for all considered species. We illustrate these methods using data about money spent managing plant invasions in different biomes of South Africa. We found clear differences between species in terms of money spent per unit area invaded, with per-unit expenditure varying substantially between biomes for some species—insights that are useful for monitoring and evaluating management. GIRAE offers a versatile and practical method that can be applied to many different types of data to better understand and manage the impacts of biological invasions.

**Supplementary Information:**

The online version contains supplementary material available at 10.1007/s10530-022-02836-0.

## Introduction

Many invasive alien species cause deleterious environmental and socio-economic impacts, with biological invasions among the main threats to biodiversity (Bellard et al. 2016; Maxwell et al. 2016; Seidl et al. 2018; IPBES 2019). Biological invasions cost the global economy tens of billions of US dollars each year – as a result of direct economic damage and money spent on damage mitigation (e.g. invader control, ecosystem restoration) (Diagne et al. 2020, 2021). Several frameworks have been developed and applied globally to conceptualise and characterise different types of impacts and costs (Blackburn et al. 2014; Jeschke et al. 2014; Kumschick et al. 2020; Van der Colff et al. 2021). Among the most highly cited papers in invasions science (see Pyšek et al. 2006 for an early review), Parker et al.’s (1999) publication (2182 citations on Google Scholar as of 2022–03–19) proposed a formula in which the total impact of an alien species is calculated as the product of its spatial extent (range size), local abundance and per-unit effect (the impact caused by a single individual, unit of biomass or unit of invaded area depending on how abundance was characterised).

The expected positive relationship between total impact and range size or local abundance is straightforward; the more individuals or the greater the invaded area, the greater the impacts. These relationships are often non-linear, with a shape varying depending on the trophic relationship between species (Bradley et al. 2019) and how these variables increase at different rates after species introduction (McGeoch and Latombe 2016; Latombe et al. 2020; Cheney et al. 2021). Per-unit effects are less well understood. Most attempts to disentangle the three components of alien species impact that account for non-linearities in these relationships have been theoretical and conceptual (Yokomizo et al. 2009; Thiele et al. 2010; Buckley and Catford 2016; Vander Zanden et al. 2017). These studies generally assume that the per-unit effect changes non-linearly with species range size and abundance, and discuss the specificities of these relationships and which factors may influence them (Strayer 2020). Other studies have proposed different variations of the decomposition of total impacts into separate components, mostly by further decomposing per-unit effects to account for various factors including the invader traits, the community composition of the invaded biota, the amount of resources and abiotic conditions (e.g. Ricciardi 2003; Strayer et al. 2006; Thomsen et al. 2011).

Despite the formula’s potential utility for comparing, ranking, and prioritising species, practical applications of the concepts that underpin Parker et al.’s (1999) formula are still missing more than 20 years after it was proposed (Wilson et al. 2020). Here, we propose the GIRAE (Generalised Impact = Range size × Abundance × per-unit Effect) approach that combines Parker et al.’s (1999) formula and more recent perspectives on the relationship between density and either impact or cost. GIRAE can thus be used to compare the components of impacts of multiple alien species using real data. We expand and linearize Parker et al.’s (1999) formula, enabling us to use linear models (and associated statistical tools) to calculate the independent components of impact (i.e., a constant per-unit effect of alien species and parameters describing the form of the relationship between impact, range size and local abundance). Better understanding of the differences in per-unit effects among species and of relationships between impact, range size, and abundance is needed to improve management decisions. Quantifying these components will also pave the way to better understand how different attributes of alien species and the environment interact to contribute to the different facets of impact (Thomsen et al. 2011).

We propose two different methods to implement GIRAE—a species-specific method and a multi-species method. The species-specific method estimates the per-unit effect and parameters for range size and local abundance for a given species based on data on range size and local abundance for different sites or regions. It is in line with existing approaches based on the density-impact relationship (Yokomizo et al. 2009; Thiele et al. 2010; Norbury et al. 2015; Strayer 2020), and allows for a detailed comparison between species. Although useful for capturing and conceptualising differences in the impacts of different alien species, the applicability of this method is limited by data availability. By contrast, the multi-species method can be applied in the context of simultaneous invasions by multiple alien species across multiple sites or regions, with each species characterised by a single combination of total impact or cost, (treated) range size, and average abundance in each invaded or managed site or region. This method assumes a similar relationship with range size and local abundance over the set of alien species under consideration; it is more feasible in practice as the required data are more readily available.

The impact formula from Parker et al. (1999) focuses on ecological impact. However, we argue that the concepts can be readily extended to other measures. As we demonstrate below, the approach can be applied to compute per-unit effects, costs, expenditure, or whichever indicator or metric is relevant, as long as the relationship with range size (or managed range size, in the case of management costs and expenditure) and local abundance monotonically increases, to compute per-unit effects, costs or expenditure.

In this paper, we use a case study of alien plant invasions in South Africa to illustrate this approach. Specifically, we combine data on the money spent controlling 18 alien plant species in nine biomes (van Wilgen et al. 2012) with data on the distribution and abundance of these species to examine how per-unit expenditure on management differ between species in different biomes, based on how resources are allocated between target taxa. In this context, GIRAE provides a method to highlight systematic differences in the amount spent controlling different types of invasions, even when total management expenditure is established a priori due to practical constraints. Finally, we discuss how the general approach introduced here is applicable to (arguably more complex) measures of socio-economic and ecological impacts, and suggest ways of overcoming limitations of the proposed methods, including data collection.

## Methods

### Theoretical concepts of per-unit effects formula: the GIRAE approach

Parker et al. (1999) introduced the following formula to relate the total impact I of an alien species to its range size R, abundance per-unit area A across range R (referred to as local abundance hereafter) and the per-unit effect E (i.e. per-unit abundance and per-unit area):

1 $${\text{I}} = {\text{R}} \times {\text{A}} \times {\text{E}}$$

The units of range size and abundance, and the type of impacts can vary. Range size can be expressed as ha or m^2^ or occupancy of particular sites; units of abundance can be numbers of individuals, biomass, or percent coverage; and units of impact might be financial or in terms of some index of environmental damage caused (e.g. biodiversity intactness or the environmental impact classification for alien taxa; see section “Discussion” for more details). If I represents the total money spent on management, R the treated range size, and A the local abundance across the treated range, then E is the management expenditure per-unit abundance and per-unit area treated (and variation in E across sites or taxa will be of interest to those monitoring and planning management).

Equation (1) assumes that I increases linearly with R and A. However, depending on how impact is characterised, the relationships are more likely to be non-linear. For example, the relationship between the abundance of alien species and native species diversity is often negative and convex (Bradley et al. 2019), i.e. there is a non-linear relationship between the abundance of alien species and the absolute loss of native species diversity, which is one possible measure of impact. This has often been interpreted as a variation in per-unit effect with R and A. That is, Eq. (1) can be reformulated as:

2 $${\text{I}} = {\text{R}} \times {\text{A}} \times {\text{E}}\left( {{\text{R}},{\text{A}}} \right)$$

**Fig. 1:**
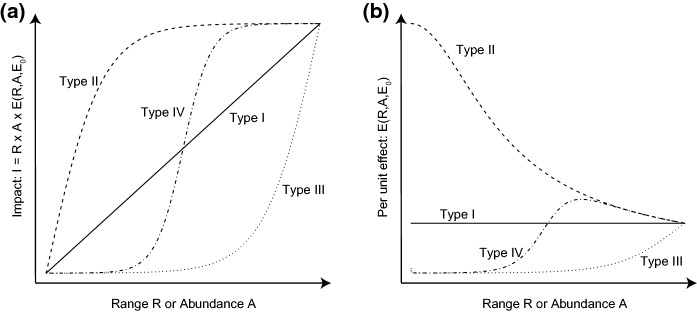
Four archetypes of relationships between total Impact (I), Range size (R), Abundance (A) and per unit effect E of an alien species following the formula proposed by Parker et al. (1999). **a** The relationship between I, R and A is often non-linear. Note that a different curve can describe the relationship between I and R and between I and A, i.e. I can increase differently with R and A. I can then be computed as the product of the values for a given (R, A) combination. **b** The non-linearity is generated by the relationship between the per-unit effect E and R, A and the constant E_0_ (as for I, the relationship can differ for R and A). The shapes of the relationship are for illustrative purposes; the exact shapes will vary depending on the system and the method used to estimate them

Four typical, theoretical relationships between I, E, R and A have been proposed to develop the concepts behind Eqs. (1) and (2) (Yokomizo et al. 2009; Thiele et al. 2010; Buckley and Catford 2016; Vander Zanden et al. 2017) (Fig. 1). Type I curves assume a linear relationship between I and R or A, and therefore a constant per-unit effect E, independent of R and A. Type II curves assume that the per-unit effect E of a species is high at low range size and abundance values, but decreases as range size and abundance increase. Type II curves therefore lead to a rapidly increasing Impact at low R and A values, which tends to saturate at high values. Type III curves assume the opposite of type II curves: per-unit effects are low at low range size and abundance, but increase as range size and abundance increase. Under type III relationships, alien species must reach a certain range size or abundance before substantial (measurable) impact manifests. Finally, type IV curves are a mix of type II and III curves, with a slow increase in impact at low R and A values, which temporarily accelerates before tending to saturate at high R and A values.

These relationships are useful for conceptualising how impacts and per-unit effects scale with changes in range size and local abundance of alien species. Although they have been modelled in different studies using a variety of approaches and combinations of variables (Norbury et al. 2015), a general approach to apply them quantitatively across species and environments has yet to be proposed (but see Cuthbert et al. (2019) for the related concept of functional response). To do so, we propose to generalise Eq. (1) as follows:

3 $${\text{I}} = {\text{R}}^{{\upalpha }} \times {\text{A}}^{{\upbeta }} \times {\text{E}}_{0}$$

where E_0_ is the per-unit effect of a species for one unit of range size and abundance; it is therefore constant for a given species, contrary to the interpretation of Eq. (2). Note that Eq. (3) can also be written as follows, to account for the fact that per-unit effects can vary with range size and local abundance, as conceptualised in Eq. (2):

4 $${\text{I}} = {\text{R}} \times {\text{A}} \times \left( {{\text{R}}^{\alpha - 1} \times {\text{A}}^{\beta - 1} \times {\text{E}}_{0} } \right) = {\text{R}} \times {\text{A}} \times {\text{E}}\left( {{\text{R}},{\text{A}}} \right)$$

The *α* and *β* exponents enable us to capture some non-linearities between total impact, range size and local abundance. In particular, we can model three of the four theoretical relationships described above (Fig. 1). Unit values for *α* and *β* indicate a linear relationship between impact, range size and abundance, i.e. a type I relationship. Values of *α* and *β* below one but greater than zero lead to a deceleration in the increase of impact with range size and local abundance, and therefore corresponds to a type II relationship, noting that this functional form does not allow impact to truly saturate, but only to decelerate as R and A increase. Values of *α* and *β* greater than one leads to an acceleration in the increase of impact with range size and local abundance, and therefore corresponds to a type III relationship. Values below 0 are not considered here because, *α* = *β* = 0 would indicate a constant impact, regardless of range size and abundance, and *α*, *β* < 0 would indicate that impact decreases with range size and abundance, which is unrealistic.

Using a log-transformation, we can linearize Eq. (3) as follows:

5 $$\log \left( {\text{I}} \right) = \alpha \cdot \log \left( {\text{R}} \right) + \beta \cdot \log \left( {\text{A}} \right) + \log \left( {{\text{E}}_{0} } \right)$$

Linearizing Eq. (3) enables us to use powerful statistical tools, including linear models and mixed effects linear models, to estimate the parameters *α* and *β*, and the per-unit effect constant E_0_, while accounting for statistical significance, as we develop below.

Although type IV relationships can be modelled mathematically by a sigmoid function as $1/(1 + \exp \left( { - \alpha \cdot \left( {{\text{R}} - {\text{R}}_{0} } \right)} \right)$, the product of sigmoid functions would not allow for a linearization after a log-transformation, as in Eq. (5), and so are not considered further here.

### Species-specific method

**Fig. 2:**
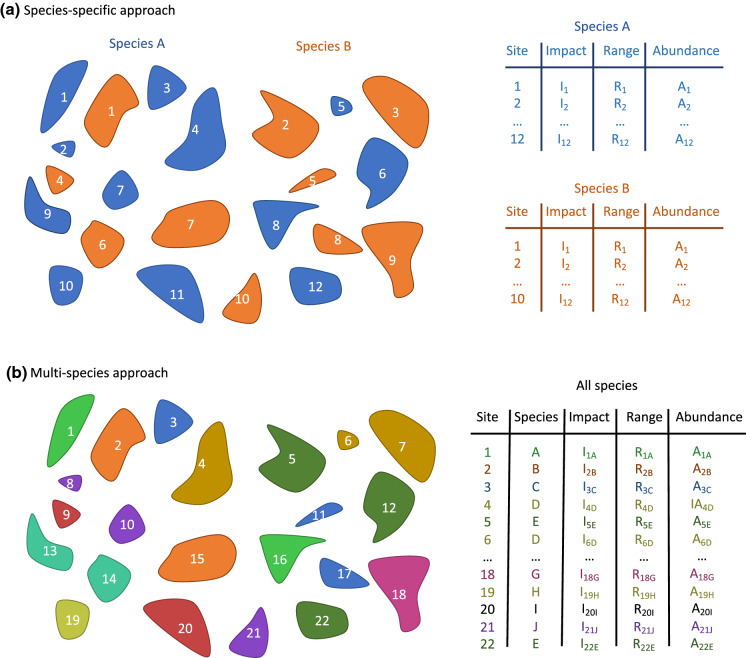
Types of data suitable for the species-specific or the multi-species approach. Colours represent different species, and numbers represent site IDs. **a** The species-specific approach can be used if there are enough populations of alien species in geographically distinct sites, and will generate different estimates of the *α* and *β* parameters from Eq. (2), as illustrated in Fig. 3. **b** If there are only a few populations for each species, all data can be combined and each separate population of species in each site can be treated as a data point, as illustrated in Fig. 4

Ideally, Eq. (5) would be applied to species individually, to obtain a unique relationship for each species. Applying Eq. (5) to a single species requires data on impact, range size and local abundance in different locations where the per-unit effect is assumed to be driven by the same factors, and therefore to have the same relationship with R and A (Fig. 2a). In that case, log(E_0_) for a given species is simply the intercept of the following linear model:

6 $$\log \left( {\text{I}} \right)\sim {\text{ log}}\left( {\text{R}} \right) + {\text{log}}\left( {\text{A}} \right)$$

**Fig. 3:**
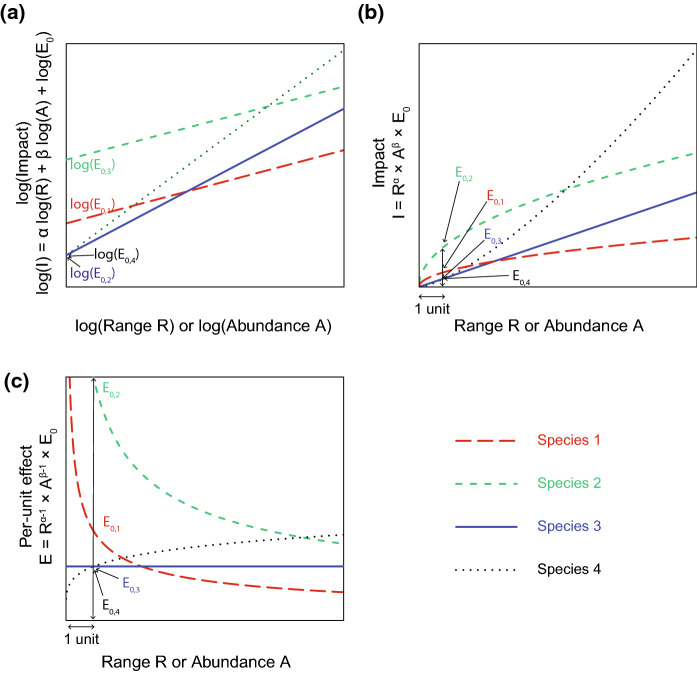
Theoretical illustration of the calculation of the relationship between impact I, per-unit effect E, range size R and local abundance A of alien species, following the species-specific method that computes the relationship for each species independently. **a** For each species, the relationship between log(I), log(R), log(A) and log(E_0_) is computed using a linear model. The *α* and *β* coefficients can take different values: between 0 and 1 (species 1 and 2; type II); equal to 1 (species 3; type I); or above 1 (species 4; type III). For each species, log(E_0_) is the intercept. Note that the values of *α* and *β* will likely be different for the same species, and a species will be characterised by a combination of two slopes, i.e. a surface, simplified here as a one-dimensional slope for clarity. **b** Exponential-transforming the relationship reveals that, for this theoretical example, the relationship decelerates for species 1 and 2, and that the lower E_0_ value for species 1 leads to a quicker deceleration. The unit value for the coefficients of species 3 leads to a linear relationship, whereas the relationship accelerates for species 4. For each species, E_0_ is the value of I for one unit of R and A. **c** These relationships can be expressed as the relationship between the per-unit effect of each species and their range size or abundance. The per-unit effect of species 1 and 2 decreases with range size and abundance, whereas it increases for species 4, and is constant for species 3

Applying Eq. (6) to individual species separately would generate comparable *α*, *β*, and E_0_ values between species, allowing us to predict when a species will have a greater or lower impact than another, and therefore to better prioritise management actions (see Fig. 3 and section “Discussion”). This approach could therefore be applied to datasets with comprehensive and comparable data on species range, abundance and impact in multiple locations, but we know of no such publicly available datasets for multiple species.

### Multi-species method

Multiple alien species often co-occur in a given area, but can have different spatial distributions within this area, and different impacts (McGeoch and Latombe 2016; Cheney et al. 2021). When each species is characterised by a single I, R and A value in a given area, these data can be combined across species to calculate a measure of per-unit effect per species (Fig. 2b). Given a set of multiple species *s*, and if we assume that the relationship between the total impact I, R and A is the same for all species included in the analyses (but see section “Discussion” for further details on this topic), Eq. (5) can be reformulated as follows:

7 $$\log \left( {{\text{I}}_{{\text{s}}} } \right) = \alpha \cdot \log \left( {{\text{R}}_{{\text{s}}} } \right) + \beta \cdot \log \left( {{\text{A}}_{{\text{s}}} } \right) + \upepsilon_{0} + \upepsilon_{{\text{s}}} = \log \left( {{\text{I}}_{{0{\text{s}}}} } \right) + \upepsilon_{{\text{s}}}$$

where $\in$_s_ is the residual for species s, after computing $\log \left( {{\text{I}}_{{0{\text{s}}}} } \right)$, the value of the following linear model for the R and A values for species s (i.e. for R_s_ and A_s_):

8 $$\log \left( {{\text{I}}_{0} } \right)\sim { }\log \left( {\text{R}} \right) + \log \left( {\text{A}} \right)$$

Therefore, ϵ_0_ is the intercept of the linear model, and can be interpreted as the baseline impact of all species included in the analysis.

**Fig. 4:**
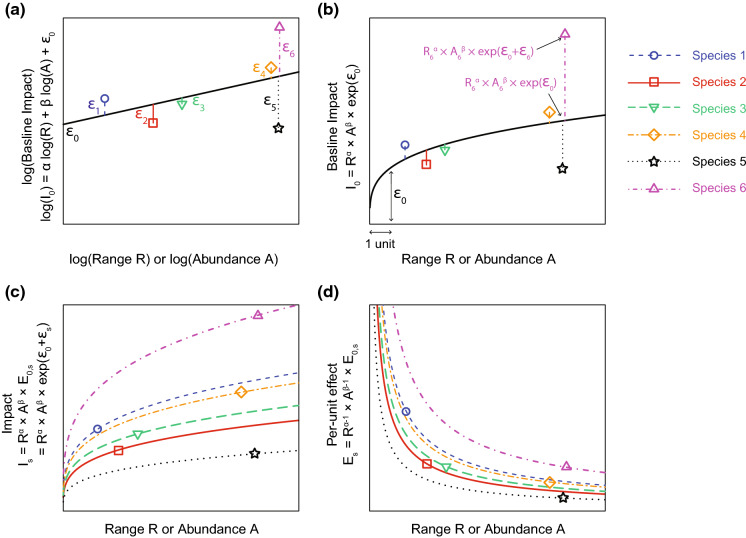
Theoretical illustration of the calculation of the relationship between impact, per-unit effect, range size and abundance of alien species, following the multi-species method that computes the relationship using data from all species simultaneously, when each species is characterised by a single (I_s_, R_s_, A_s_) combination, contrary to the species-specific method (Fig. 2). **a** The relationship between log(I_0_), log(R), log(A) and log(E_0_) is computed using a linear model based on the combined data from all species. As a result, only one combination of *α* and *β* coefficient values, and a single intercept ϵ_0_, are computed for all species. Note that a combination of *α* and *β* values would general a two-dimensional surface, simplified here as a one-dimensional slope for clarity (with a slope in [0, 1] for the sake of the example, but it can be above 1). The resulting I_0_ variable represents the baseline impact across species. The difference between the logarithm of the impact of each species log(I_s_) and log(I_0_) is indicated by the residual values ϵ_s_. **b** The relationship can be exponential-transformed to reveal how the observed impact values I_s_ differ from the baseline I_0_. Since the *α* and *β* coefficient values are below 1 in this example, the baseline I_0_ decelerates as R and A increase. **c** A relationship is computed for each species, which only differ from each other in the E_0,s_ values. Since all species share the same α and β values, the I_s_ values decelerate as R and A increase for all species. **d** These relationships can be expressed as the relationship between the per-unit effect of each species and their range size or abundance, which decreases for all species

The logarithm of the per-unit effect of each species s, log(E_0,s_), corresponds to the sum of the intercept ϵ_0_ plus the residuals ϵ_s_ after fitting Eq. (8), as shown in Eq. (9) (Fig. 4).

9 $$\log \left( {{\text{E}}_{{0,{\text{s}}}} } \right) = \upepsilon_{{\text{s}}} + \upepsilon_{0} = \log \left( {{\text{I}}_{{\text{s}}} } \right) - \log \left( {{\text{I}}_{{0{\text{s}}}} } \right) + \upepsilon_{0}$$

### Impact or cost vs. pre-determined expenditure

**Fig. 5:**
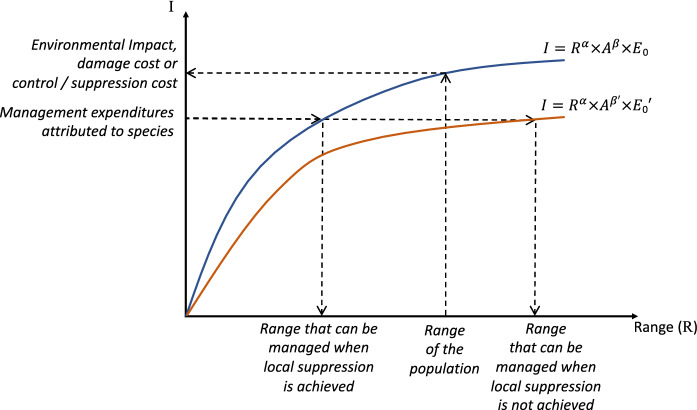
Difference in the causal relationship between the measure of impact or cost and Range on the one hand, and management expenditure and Range on the other hand. For impact or cost, I is determined by the range and local abundance of the species, as represented by the upward and leftward arrows, assuming the relationship follows the blue curve. When management expenditure is determined a priori and below control or suppression cost, the range and local abundance that can be managed will depend on the expenditure, as represented by the rightward and downward arrows. We can distinguish two main scenarios. Under the first scenario, the alien species population are suppressed in each visited location (i.e. the abundance that is managed is the local abundance). The relationship between I, R and A will then be the same as for suppression cost (represented by the blue curve), E_0_ represents the per-unit suppression cost, but not all the range covered by the alien species can be managed (left downward arrow). Under the second scenario, the alien species is not successfully suppressed in the visited locations (i.e. the abundance that is managed is lower than the local abundance). The value of per-unit management expenditure is then lower than the per-unit suppression cost (E_0_′ < E_0_), and a wider area can be treated (right upward arrow), although not as efficiently, as represented by the fact that the orange curve is below the blue one. The value of the β’ parameter will depend on the difference between the local abundance and the number of treated units. The difference in the direction of causality or the local management success do not change the fact that the relationship between these elements and per-unit effect can be assessed using the same general parametric form represented by Eq. (2)

Equation (1) was developed with ecological impacts in mind, but is also appropriate for considering other metrics, e.g. economic impacts or management costs, whose value increases with range size and local abundance (Parker et al. 1999). Our generalisation [Eq. (3)] can therefore encompass multiple types of effect, including the economic impacts of invasions and the amount spent on their management. From a causal relationship, two types of measures of impact and cost can be distinguished. First, as initially discussed by Parker et al. (1999), impacts and damage costs are the consequences of invasion, and will be higher if an invasive species is more abundant and covers a wider area (Fig. 5, blue curve). Control cost also depends on the cost of managing one individual or unit of biomass, and on the range size and local abundance treated. In this study, control cost is the cost necessary to reduce a population of an alien species below a management threshold, i.e. its ‘suppression’ sensu Robertson et al. (2020). We thus use ‘control’ as a general term that encompasses different types of management beyond suppression (see Robertson et al. (2020) for a range of management types), and ‘suppression’ when referring to the type of control considered in this study. When the goal of management is the suppression of a population of alien species, the larger the area to manage, the costlier it is likely to be to pursue management, as management actions involve moving people and equipment. Similarly, the more abundant a species is at a given location, the costlier it is to suppress, because of the increase in time required (although this relationship will depend on the management approach used, with notable differences between treating individual plants e.g., through cut-stump herbicide application, versus treating an area, e.g., through broadcast aerial application of herbicide). These relationships can then accelerate or decelerate (i.e. increasing or decreasing per-unit management cost, Eq. (4), Fig. 1), depending on the logistics and approaches used to manage the alien species, the existence of fixed costs (e.g., start-up costs), etc., and will of course likely be different for ecological impacts, damage costs or control or suppression costs.

The amount of money spent on the management of a particular species (i.e. management expenditure) is nonetheless often decided a priori, and is influenced by many factors (Panetta 2009). As a result, the causal relationship is inverted, as the range and abundance that can be managed will be determined by the management expenditure (Fig. 5). E_0_ then represents the money spent per unit of alien species. If species are successfully suppressed at each treated site, E_0_ amounts to the per-unit suppression cost (technically the per-unit extirpation cost as it assumes an abundance of zero), and the *α* and *β* coefficients will have the same value, even if management expenditure is lower than suppression cost (Fig. 5). If suppression (or extirpation) is not achieved, E_0_ will necessarily be lower than the per-unit suppression cost. The value of the *α* coefficient (for range) will remain the same (it will depend on the same logistics constraints of moving between sites), but the value of the β coefficient (for abundance) will depend on the difference between local abundance and the number of treated units of alien species (e.g. it would be 0 if the same number of individuals are removed at each site).

Nonetheless, even if management expenditure is set at a lower level than control costs and management does not lead to suppression, the relationship between I, R, A and E_0_ can be considered to follow the same general parametric form, given by Eq. (3). In other words, assuming the relationship between I, R, A and E_0_ can be correctly assessed from Eq. (6) or Eq. (7), data on I, R and A will enable to estimate E_0_ regardless of their value and of the meaning of I and E_0_.

### Data

To estimate the per-unit effect of multiple species, GIRAE requires unambiguous data on impact (or alternatively cost or expenditure), range and abundance for these species. Such data is scarce in public repositories. Here we combined data from multiple sources for South Africa, using management expenditure as I, to illustrate the approach since, as we develop above, the relationship between management expenditure, range size and local abundance can be captured by GIRAE. Note that we do not consider that management expenditure is an indicator of impact equivalent to damage cost or environmental impact.

#### Management costs across sites and taxa

Using South Africa as a case study, we explored the available data on management costs compiled in InvaCost, the most comprehensive database on economic costs of alien species worldwide, which considers both damage and management costs (Diagne et al. 2020). InvaCost offers the advantage in that it clearly attributes an unambiguous measure of cost to each species separately in a given country, which is appropriate to apply our approach. InvaCost lists 31 studies in South Africa, which provided a comprehensive overview of the economic data available. Amongst these studies, due to issues of comparability, only van Wilgen et al. (2012) provides comparable estimates of the amount of money spent managing 18 invasive alien plant taxa (species, genera or families) in nine biomes in South Africa by the Working for Water program between 1995 and 2008, in South African Rands. We therefore use these data to demonstrate the two approaches outlined above.

#### Species distribution

Fitting Eq. (8) requires data at fine scale on range size and abundance of alien species over a large region. van Wilgen et al. (2012) reports the estimated occupied area by these species per biome in 1996 and 2008, obtained from three independent studies (Le Maitre et al. 2000; Kotzé et al. 2010; Van den Berg et al. 2013), and the area treated by the Working for Water program between 2002 and 2008 (all expressed in condensed area, i.e. the treated area effectively occupied by the species, rather than the total extent of occurrence over which the species was managed; see section “Discussion” for details about different measures of range size). We used the area treated as a measure of range size in Eq. (8).

For abundance data, we used estimates from the Southern African Plant Invaders Atlas (Henderson 2007; Henderson and Wilson 2017). Spatially explicit abundance data at the scale of a country is scarce, and SAPIA is the most comprehensive database on alien plant species distribution in South Africa. It comprises records gathered by 670 participants since 1994, in addition to road surveys by the lead author since 1979 over the whole country (we only kept surveys prior to 2008, which is the latest year of the Working for Water assessment reported in van Wilgen et al. (2012). SAPIA records are, with the exception of the earliest records, geo-referenced to point localities, but notes were taken as to the abundance of invasive plants at the landscape scale (and so at resolutions in the range 1–10 km). Abundance was scored as present: abundance unknown or not recorded; rare: one sighting of one or a few plants; occasional: a few sightings of one or a few plants; frequent: many sightings of single plants or small groups; abundant: many clumps or stands; and very abundant: extensive stands. Records whose abundance was scored as present were removed from the analyses since it can belong to any of the other categories. We converted the remaining five classes to an exponential scale [exp(1) to exp(5)] assuming that increases in abundance would be similar to those described in (Wilson et al. 2018) for four categories (< 2%, 2–10%, 10–50%, > 50%). Note that these values [exp(x)] need not represent the actual number of individuals or biomass units; only their relative values are required, as discrepancies with actual values will be accounted for by parameter β in Eq. (5) (see the section “Discussion” for further details on this topic). For each record, we extracted the biomes in which it was recorded based on its coordinates and the 2006 vegetation map of South Africa, Lesotho, and eSwatini (previously Swaziland) (South African National Biodiversity Institute 2006), as 2006 is encompassed in the period over which alien species were managed and should therefore best correspond to the biomes reported by van Wilgen et al. (2012).

Many factors come into play when selecting invaded areas to be treated, including the level of invasion, accessibility, native biota, etc. (Roura-Pascual et al. 2009, 2010). We lacked information on how management sites were selected in our case study. Therefore, instead of using the average abundance over all records as our measure of abundance in Eq. (5), we sampled records for each species and biome, using three different sampling approaches, to assess the sensitivity of the results to site selection. We sampled records based on the proportion Parea_S,B_ of treated area over the total area occupied by the species over the 1996–2008 period. The total occupied area was calculated as the maximum of the area occupied in 1996 (i.e. before management) or the area occupied in 2008 plus the area treated between 2002 and 2008 (i.e. the area after management plus the treated area), as reported by van Wilgen et al. (2012) (as natural growth of alien species still occurs during treatment, area in 2008 could be more important than area in 1996 despite treatment, in addition to the fact that areas were estimated differently in the two studies). Under the first sampling approach, we assumed management effort was randomly allocated across invaded sites, so for each species and biome, Parea_S,B_% of the records were sampled randomly [restricting the selection to the Northern Cape province for *Prosopis* species, as indicated in van Wilgen et al. (2012)], and the average abundance of the records was reported. Under the second approach, we assumed that management prioritised areas that were densely invaded, as alien species should have the highest ecological impact in such sites (Shackleton et al. 2017). Observed abundance of records (exp(1) to exp(5)) was used as a weight when sampling, and reported the average abundance over the records selected using this weighted sampling. Under the third approach, we assumed that the least invaded areas (i.e. those with sparse stands of invaders over large areas) were more likely to be treated, as such sites would be more easily managed and more likely to limit invasive spread, leading to greater management efficacy. The inverse of the observed abundance of records (exp(−5) to exp(−1)) was used as a weight when sampling, and reported average abundance. We only used these three sampling approaches to assess the sensitivity of the results, due to the lack of information on site selection, but other criteria could be applied (Shackleton et al. 2017).

### Calculation of the per-unit management expenditure

Since each species has only between one and five management costs in the focal dataset, it is not possible to apply the species-specific approach for each species separately on the case study. Rather, this dataset is more appropriate for the multi-species approach, to estimate a per-unit expenditure for each species-biome combination. We nonetheless demonstrate how to apply the species-specific approach by combining records for all *Acacia* species together, as they represent about a third of the data (10 out of the 33 species-biome expenditure values), and comparing results to all other species combined. We also demonstrate how to apply the multi-species approach using data for all species together.

Based on the random samples described above, we applied linear models using the base lm() function from R v4.0.3 (R Core Team 2022), to compute the coefficients of Eqs. (6) and (8) from the 33 data points corresponding to the species by biome combinations. We quantified I as half of the management expenditure between 1995 and 2008 (since it represents twice the period over which the extent of the treated area was provided). R was quantified as the condensed treated area per species and per biome, and A as the mean abundance in the sampled records. The per-unit management expenditure E_0_ of the different species in the biomes they were managed were then calculated using Eq. (9).

Random sampling of records and regressions was performed 1000 times for each sampling approach (random or weighted sampling), selecting Parea_S,B_% of the records each time. That means that between replicates, I and R are constant, but A varies; consequently, the approach generates a distribution of E_0_ values that reflects the variance of abundance between records for each species × biome combination. Normality of the residuals was assessed using Q–Q plots (Figs. S1–S6).

## Results

### Species-specific approach

The median variance explained by the linear model over the 1000 replicates was within [0.92–0.94] for *Acacia* species, and within [0.81–0.83] for other species (adjusted r^2^, Figs. S7–S9, slightly higher for management weighted by local abundance). *α* coefficients for range size were always significant (*p* values < 0.01). By contrast, only 8.9, 13.6 and 4.5% of *β* coefficients for abundance were significant across replicates for *Acacia* species, and only 0.7, 0 and 6.3% for other species (*p* values ≤ 0.05; results are presented for the random allocation of management, management weighted by local abundance of SAPIA records, and management weighted by the inverse of local abundance of SAPIA records respectively). Coefficients were always below 1 for range size (median values were 0.72, 0.73 and 0.72 for *Acacia* species and 0.7, 0.66 and 0.71 for other species), indicating a decelerating relationship with management cost (type II relationship, Fig. 1). 52.5, 71.1 and 47.2% of the coefficients for abundance were above 0 for *Acacia* species, and 16, 99.2 and 2.5% for other species (median values were 0.03, 0.32 and −0.02 for Acacias, and −0.1, 0.17 and −0.23, for other species; all values were below 1). None of the values below 0 was significant. Overall, these results indicate a substantial relationship between management expenditure and range size, but a very weak relationship for abundance (Figs. S10–S12). The median per-unit expenditure was 13.3, 3.6 and 15.2 for *Acacia* species depending on the sampling approach, and 16.7, 6.2 and 19.6 for the other alien species, with 70.7, 24.5 and 97.1% of significant values for *Acacia* species and 100, 66.6 and 100% for other species (*p*-values ≤ 0.05).

### Multi-species approach

The median variance explained by the linear model over the 1000 replicates was 0.86 (adjusted r^2^, Figs. S13–S15., slightly higher for management weighted by local abundance). *α* coefficients for range size were always significant (*p* values < 0.001). By contrast, only 0.3, 7.6 and 5% of *β* coefficients for abundance were significant across replicates (*p* values ≤ 0.05; results are presented for the random allocation of management, management weighted by local abundance of SAPIA records, and management weighted by the inverse of local abundance of SAPIA records respectively). Coefficients were always below 1 for range size (median values were 0.69, 0.68, 0.7), indicating a decelerating relationship with management cost (type II relationship, Fig. 1). 41, 97.1 and 12.7% of the coefficients were above 0 for abundance, and all values were below 1 (median values were −0.02, 0.2, −0.13). None of the values below 0 was significant. Overall, these results indicate a strong relationship between management expenditure and range size, but a very weak relationship for abundance (Figs. S16–S18).

**Fig. 6:**
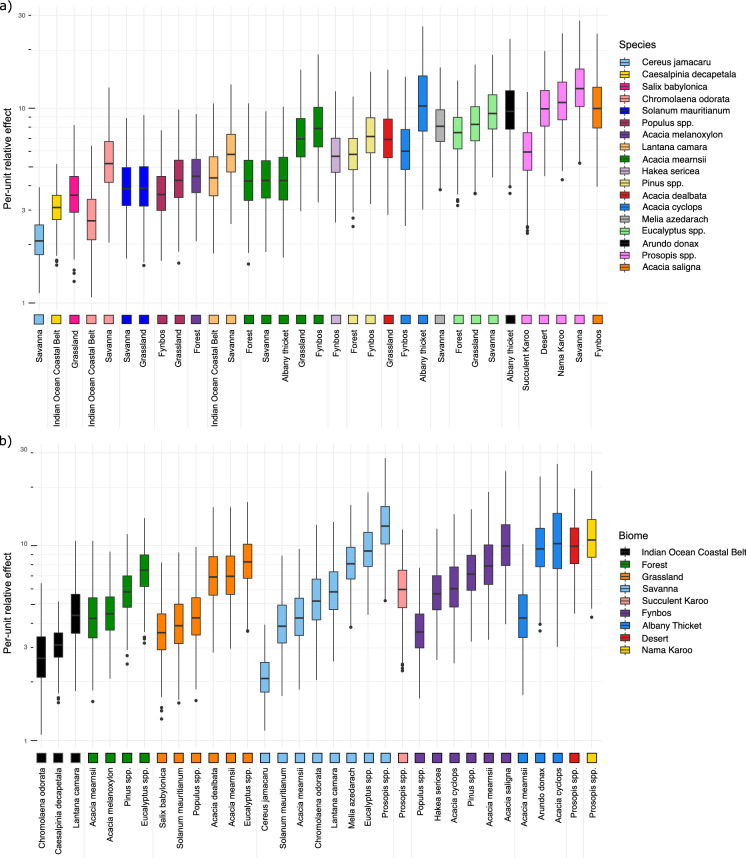
Distributions of per-unit relative effects (here money spent per-unit on management) of invasive plant species managed by the Working for Water program in South Africa between 1999 and 2008, in the different biomes of South Africa, over the 1000 replicates, calculated after sampling predominantly records with high abundance. Money spent per-unit should be interpreted in a relative rather than absolute fashion, due to the lack of absolute meaning for the abundance values. **a** Species are distinguished by different colours and ordered by their median money-spent per-unit over all biomes where they were managed. **b** Biomes are distinguished by different colours and ordered by their median per-unit cost over all managed species they contain

We only report money spent per-unit for positive *α* and *β* coefficients, as negative values should be taken as artefacts from stochastic sampling. The money spent per-unit obtained after applying the multi-species method was qualitatively similar across the different approaches for allocating management effort. Since the models fitted from using the weighted sampling of the SAPIA records generated a very slightly higher explained variance, and coefficients were more consistently positive and significant than for the other two approaches, we will present these results in the following (Fig. 6, but see Figs. S19 and S20 for the money spent per-unit calculated with the other two approaches to allocating management).

The relative money spent per-unit on the different species (Fig. 6a) differed by five orders of magnitude between invasions where the least money was spent (*Cereus jamacaru* in Savanna) to those where the most money was spent (*Prosopis* species in Savanna). Some taxa showed differences in the money spent per-unit in different biomes, for example, the money spent per-unit on *Chromolaena odorata* was about twice as high in the Savanna than in the Indian Ocean Coastal Belt biome. The money spent per-unit on *Acacia mearnsii* was ~ 1.5 times higher in the Fynbos and Grassland biomes than in the Albany thicket, Forest and Savanna biomes. The money spent per-unit on *Acacia cyclops* was also ~ 1.5 times higher in the Albany thicket biomes than in the Fynbos. The money spent per-unit on *Prosopis* species was ~ 1.5 times higher in the Desert, Nama Karoo, and Savanna biomes than in the Succulent Karoo. The biomes in which money spent per-unit was higher were therefore not consistent across taxa. We observed no substantial differences of money spent per-unit across biomes overall, all taxa considered (Fig. 6b).

## Discussion

### Generalising and interpreting the calculation of per-unit effects for different types of impacts

The application of GIRAE shows that a straightforward regression approach facilitates the calculation of per-unit effects of alien taxa, thereby also disentangling contributions of local abundance and range size to total impact, cost and management expenditure. Management of protected areas simultaneously invaded by multiple alien species is often area-, rather than species-based (Cheney et al. 2021). However, area-based approaches can be less efficient for specific high-priority species that need to be targeted (Foxcroft et al. 2009). Applied to per-unit control costs, our approach can complement area-based approaches and inform management from an ecological and economic perspective. At similar abundances and range size, a species with a lower per-unit control cost E_0_ should be cheaper to manage. From a species-based management perspective, it might therefore be advantageous to manage priority species with high per-unit control costs or impacts that occur at low abundance and/or over narrow ranges, before they start spreading. This is especially true if funds for management are limited and determined a priori. If management expenditure are set up below control costs, alien species populations will likely recover after incomplete management.

To illustrate our approach, we focused on money spent on managing species. The case study presented here reflected data availability: money spent is routinely documented and such data are available for a wide range of species across different regions and across different countries (Diagne et al. 2020, 2021). However, money spent is not necessarily correlated with metrics of damage nor of ecological impact. The amount of money spent is indeed often determined independently of ecological impacts, as it is affected by feasibility, return on investment, and, in the case of Working for Water, where it is most important to create jobs in order to alleviate poverty (van Wilgen and Wannenburgh 2016). In this context, it would be important to know if alien species are effectively suppressed, to determine if the per-unit management expenditure amount to per-unit suppression cost (Fig. 5). To do so, it may be necessary to conduct surveys following management actions to assess suppression success and obtain better estimates of differences in suppression costs of different species and improve management.

The approach can be extended to other measures of expenditure (e.g., work time spent for alien species management) or impact types (e.g., damage costs, human welfare, health or ecological impacts), noting that alien species can impact multiple sectors of society, each with their own way of valuing goods and services, and different types of impacts will likely be more consistently documented than others (Diagne et al. 2020, 2021).

Environmental and social impacts will be more challenging to integrate in the formula, due to the complexity of these concepts and the paucity of data for assessing impacts at different spatial scales in these domains. Multiple measures of impact can be used, at the population, community or ecosystem level, and their suitability can vary depending on the life forms of the alien species (Norbury et al. 2015). At the community level, measures of species richness, species diversity, or species evenness have been used (Pearson et al. 2016; Bradley et al. 2019). In this context, the spatial scale at which total impact is measured is usually the same as the scale at which the range and abundance of alien species is measured. At the ecosystem level, scales may differ. For example, invasive alien trees are associated with reduced water flows in South Africa, which has been a prime motivation for the Working for Water programme (Le Maitre et al. 2002, 2016). In this context, total impact would likely be measured at the scale of a catchment, which could be larger or narrower than the scale at which the alien species is distributed. If it is larger, the range and abundance of the alien species should correspond to the whole distribution. If it is narrower, it would be more appropriate to only use the range and abundance of the alien species within the catchment. For socio-economic impacts, the scale may also differ between impact vs. range and abundance. For example, impacts on water flows may have socio-economic impacts through fishing activities, and catchment area would also be an appropriate scale to measure socio-economic impacts. The choice of scale will be context-dependent, and based on the specific type of impact that is considered.

Global classification of impact into ordered categories, such as the IUCN’s EICAT scheme (Blackburn et al. 2014), or the SEICAT scheme (Bacher et al. 2018), offer avenues to compare the impacts and per-unit effects of species from different life forms. Although EICAT and SEICAT scores for a species cannot be directly used in the approach, since they are global measures based on literature reviews, the definitions of impact categories can be of use to define impact within a given area (ranging from minimal [no noticeable impact], to minor [reduction of fitness for individuals of at least one native species], moderate [population decline of at least one native species], major [reversible local extinction of at least one native species] and massive [irreversible local extinction of at least one native species]). However, the use of ordered categories in linear and generalised linear models is not straightforward (Guisan and Harrell 2000). This analytical framework therefore represents a basis from which to develop more specific statistical tools to integrate different types of data. We also hope it will incentivise systematic collection and harmonisation of impact data with data on range size and local abundance. Doing so will allow the impacts and costs of biological invasions to be assessed, ranked and fed into decision making (many forms of analysis are routinely used as inputs into decision making; (McGeoch et al. 2016)). We discuss below different aspects related to range size and abundance that are important to consider when collecting data.

The time scale of impacts varies across species; some impacts occur very quickly whereas others can take decades or longer to manifest, leading to a slow accumulation of “invasion impact debt” (Rouget et al. 2016). That means that E can be considered as an increasing function of time t since introduction. Alternatively, recipient environments may develop defence mechanisms over time and impacts of alien species may decrease over time. E would then be a decreasing function of time since introduction. To account for such time lags, time since introduction could therefore be incorporated into Eq. (3) as follows:

10 $$\begin{aligned} {\text{I}} & = {\text{R}} \times {\text{A}} \times {\text{t}} \times {\text{E}}\left( {{\text{R}},{\text{A}},{\text{t}}} \right) \\ = {\text{R}} \times {\text{A}} \times \left( {{\text{R}}^{\alpha - 1} \times {\text{A}}^{\beta - 1} \times {\text{t}}^{\gamma - 1} \times {\text{E}}_{0} } \right) \\ = {\text{R}}^{\alpha } \times {\text{A}}^{\beta } \times {\text{t}}^{\gamma } \times {\text{E}}_{0} \\ \end{aligned}$$

where *γ* determines how *E* increases (*γ* > 0) or decreases (*γ* < 0) in time.

Abundance is not a straightforward variable to monitor in a comparable fashion for multiple species, especially for plants (Catford et al. 2012). In absolute values, abundance can be measured as numbers of individuals, cover area, or unit of biomass. Number of individuals is a suitable metrics for large animals, but biomass may be more appropriate for small animals such as insects. In the case of plants, number of individuals may be more intuitive for large trees, whereas area covered is more appropriate for herbaceous plants (Wilson et al. 2014). Relative abundance (i.e. compared to the total abundance over all species present at a site) may also be appropriate, especially in relationship with ecological impacts on other species. For example, Wilson et al. (2018) propose the following definition for each class: “Invasive plants cover *x*—*y*% of the area covered by plants, or invasive species make up *x*—*y*% of the biomass of the area; populations of invasive animals make up *x*—*y*% of all individual animals at the site.” Such categories (similar to those we used here) offer a convenient way to combine these different quantities for multiple taxonomic groups, although they imply that the per-unit effects computed with our method should also be interpreted in a relative rather than absolute fashion. This also implies that for results to be comparable across different regions, the same classification scheme should be used.

Although more straightforward than abundance, range size in Parker et al.’s (1999) formula [Eq. (1)] can also be computed in different ways. Two main measures are typically used in conservation science across taxonomic groups: the extent of occurrence (EoO) and the area of occupancy (AoO) (IUCN 2001) (but see Hui et al. (2011) for more complex alternatives). The EoO is the minimum continuous area encompassing all sites of occurrences of a taxon (for example represented by a convex hull). However, the EoO may not be appropriate when a species is introduced to a country or region at multiple locations that may be far apart leaving large areas with no individuals (and therefore no impact) in between. The AoO is the area effectively occupied by a taxon, and corresponds to the condensed area used by van Wilgen et al. (2012) and in our analyses. In practice, accuracy of the AoO depends on the spatial grain at which it is estimated. The choice of a measure for computing the per-unit effect of an alien species will be case-dependent, based on the type of impact and the context. For example, in the case of management costs, if the same team is to suppress an alien species population, it may be costlier to suppress a patchy population spread over a larger area than a population with the same abundance occurring over a small area, due to the time necessary to move from one invaded location to another. In that case, the EoO provides additional information not captured by the AoO.

### Predicting alien species impacts

Disentangling the three components of impact and characterising alien species by combinations of the three coefficients (*α*, *β*, E_0_) is an essential first step towards identifying which species and environmental attributes determine the values of the three coefficients. Doing so will enable prediction of potential impacts or future management costs of newly introduced species or species on watch lists. For ecological impacts, the predictors linked to per-unit effect can be related to the constituents of the environment, i.e. attributes of the invader, attributes of the resident species, resources levels, and abiotic conditions (Thomsen et al. 2011); they can also be attributes of the interactions between these constituents, i.e. propagule and colonisation pressure, functional distinctiveness, environmental tolerance, interactions with resident species, disturbance, and environmental heterogeneity (Ricciardi et al. 2013). Some of these predictors might be ‘universal’ (sensu Thomsen et al. 2011), i.e. readily comparable across studies invaders and habitats, or might vary with context (‘unique’, sensu Thomsen et al. 2011). Disentangling generality and mechanistic context-dependence, while accounting for apparent context-dependence that can be caused by confounding factors, sampling differences or statistical inference issues (Catford et al. 2022), will be crucial to draw generalisations and to make predictions about future impacts. In the context of invasive alien plants, for per-unit management costs, the attributes of the species are important (e.g. a large tree may be more expensive to remove than a shrub; and resprouting species are usually more difficult to suppress than those that do not resprout, etc., although post-hoc we could not detect any such effects in our analyses). The treatment that is applied (e.g. mechanical removal versus herbicide application), the terrain and accessibility of the location where populations occur, etc., should also be considered (Panetta 2009).

Different combinations of predicted range size, local abundance, *α* and *β* coefficients, and E_0_ values then imply different levels of risk. For example, for a given maximum impact I, a species with a type III relationship (*α* or *β* above 1) will only be of concern if range size is predicted to be large or local abundance high, as the total impact would remain low at low range size and abundance. In terms of management costs, for type III relationships, controlling a species will be proportionally less costly if they have not spread, as costs accelerate with range size. For ecological impacts and damage costs, a type III relationship means that mitigation approaches to keep abundance and range under the range of values impact is accelerating are likely to be the most cost-effective approaches. In contrast, for species with a type II relationship (*α* or *β* below 1), the predicted range size or abundance has little importance above a certain value (since impact mostly varies at low R or A values), and concern will be mostly determined by the per-unit effects of species and the resulting maximum impact they can have. In our analyses on the control of invasive alien plants, we observed a type II relationship between management expenditure and range size, computed as the area of occupancy. This might be due to the fact that within a given region with fixed boundaries, the extent of occurrence will mathematically saturate quicker than the area of occupancy, but might contribute more to management expenditure linked to the displacement of people and equipment across invaded sites. In that case, it might be more cost-effective to manage priority species with a high AoO/EoO ratio.

Overall, the sign of the *α* and *β* coefficients will be influenced by different factors linked to the type of impact considered, to the life form of the alien species considered (e.g., plants/mammals/insects, terrestrial/freshwater/marine species, etc.), and to the context, such as the presence or absence of other species (Catford et al. 2022). For example, the trophic levels of native and alien species is likely to influence the relationship between ecological impact and abundance, with predator alien species likely displaying a type III relationship, but relationships would be more variable for alien species at the same trophic level as the impacted native species (Bradley et al. 2019). The presence of an apex predator can also decrease the impact of mesopredators at similar abundances through the suppression of their activity (Feit et al. 2019). For range size, the type of relationship might depend on connectivity and the relative propagule exchanges of native and alien species. For management costs, the sign of the *α* and *β* coefficients will likely be influenced by logistics, including the transport of equipment across space for range size, but also the ecology of the alien species. For example, some plant species develop deep root systems, making them harder to remove the longer they have been invasive at a site.

### Limitations of the approach and solutions

Here, management expenditure was reported at the scale of biomes, and some species were managed in one or two biomes. However, effects can be assessed at other spatial scales. Spatial units of assessment can encompass lakes (Latzka et al. 2016), patches of several thousands of hectares (Holland et al. 2013), 100 × 100 m sites (Pearson et al. 2016), etc. Fine-scale assessments of impacts at multiple locations, congruently with local abundance and area of the location, recorded for multiple species, would allow for a broader application of the species-specific method. Different types of impacts would likely be measurable at different spatial scales, and results should be interpreted in light of the scale.

The multi-species method generates a fixed per-unit money spent E_0_ for a species in a given environment, that is independent of its abundance and range size. The approach assumes that the relationship between money spent or impact and range size or local abundance is the same for all species in the different biomes. As a result, E_0_ represents the per-unit money spent of a species in the absence of conspecifics (i.e. when it is only present in a unit of range size by a unit of abundance), therefore allowing comparisons of alien species irrespective of their abundance or range size. Applying the multi-species approach is a necessary simplification, as comprehensive data on species range, local abundance and money spent or impact in multiple locations required for the species-specific method are missing for most species. The high variance explained by the multi-species model and the results from the species-specific analyses nonetheless suggest that this is a valid approximation in our case study on money spent per species in different biomes. The variance explained was higher for *Acacias* than for the other species grouped together, which was expected since the techniques used to manage invasions by *Acacia* species are similar (Wilson et al. 2011). However, the *α* and *β* coefficients and the per capita effects of the other taxa were of similar orders of magnitude.

If data are not available at the species level, but different species are expected to be characterised by different relationships between impact, abundance and range size, a compromise would be to apply Eqs. (6)–(8) to different groups of species, like we did to illustrate the species-specific approach. Groups should be established so that the relationship between per-unit effect, range size and abundance can be assumed to differ between groups, but to be the same within groups. Grouping species according to these relationships should be guided by expert knowledge on the biology of the species (e.g. invasion syndromes; Perkins and Nowak 2013; Novoa et al. 2020)). In our example, grouping Acacias together is more coherent than grouping all other species together, which is indeed reflected by the difference in variance explained.

Difficulties to disentangle the impacts of different species will arise when multiple alien species are invading the same area. Pearson et al. (2016) addressed this issue by incorporating multiple invader in the same linear model assessing total impact, measured as a decline in native species abundance in plots of equal size, as $I = A_{species} \times E_{species} + A_{others} \times E_{others}$, where A_species_, E_species_, A_others_ and E_others_ are the abundance and per-unit effect of the focal invader and other alien species, respectively. They thus considered that impacts from multiple alien species are additive. In their model, per-unit effect is the coefficient estimated with the linear model. However, their model only accounts for species abundance, and does not consider non-linear relationships. In GIRAE, the presence of two (or more) invaders simultaneously could be accounted for by considering impacts to be multiplicative, as follows:

11 $${\text{I}} = {\text{R}}_{1}^{{{\alpha }_{1} }} \times {\text{A}}_{1}^{{{\beta }_{1} }} \times {\text{E}}_{0,1} \times {\text{R}}_{2}^{{{\alpha }_{2} }} \times {\text{A}}_{2}^{{{\beta }_{2} }}$$

12 $$\begin{aligned} \log \left( {\text{I}} \right) & = \alpha_{1} \cdot \log \left( {{\text{R}}_{1} } \right) + \beta_{1} \cdot \log \left( {{\text{A}}_{1} } \right) + \alpha_{1} \cdot \log \left( {{\text{R}}_{2} } \right) \\ \quad + \beta_{2} \cdot \log \left( {{\text{A}}_{2} } \right) + \log \left( {{\text{E}}_{0,1} \times {\text{E}}_{0,2} } \right) \\ \end{aligned}$$

However, this approach has important limitations. In addition to the fact that impacts may very well not be multiplicative, Eqs. (11) and (12) cannot directly disentangle the per-unit effects of multiple co-occurring alien species. Rather, it may be possible to apply the multi-species approach, considering each combination of species as a separate element in Eq. (7), and to then compare the per-unit effects of different combinations of species to better understand how they add up. This will be a complex task, requiring more in-depth work.

### Money spent managing invasive plants in South Africa

Our results show a strong relationship between management expenditure and occupancy, probably reflecting logistic constraints of moving from one invaded location to another. For abundance, the β coefficient were close to zero and mostly non-significant. That suggests that management expenditure is mostly independent from local abundance, which may be due to a range of factors, including the technique used for treating the area. For example, it can take much greater effort to detect and reach sparse invasions compared to the relative ease (per individual plant) of treating dense infestations.

We found that species identity played an important role in explaining the money spent per-unit. Differences in per-unit money spent between biomes were not consistent across the taxa present in multiple biomes. This was probably due to the spatial grain of biomes being too large, and each therefore encompasses a wide variety of terrains and conditions which influence money spent per-unit. Nonetheless, the clear differences between the money spent per-unit on the same taxa in different biomes suggests that the differences in protocols and methods used for their management in different biomes warrant further investigation. This may be due to genuine differences in the ease of management of particular species between biomes, reflect fundamental differences in the relationships between abundance and range size in different environments, emerge from the simultaneous management of multiple alien species in the biomes, or reflect differences between contractors and personnel expertise.

It is important to note that we did not have data on the outcome of the management, i.e. whether the money spent on control was effective in reducing the invasive plant populations. Our analysis looked only at broad-scale inferences of occupied areas before and after the Working for Water assessment, which were performed using different methods. As such, the comparisons as described above are in terms of per-unit expenditure rather than control or suppression costs (i.e., how much it would cost to keep an invasion below a threshold). Explicit assessments of management efficacy are rare in South Africa (Zengeya and Wilson 2020), although see e.g. (Kraaij et al. 2017). In other words, our analyses reveal variation in how money is spent across species and biomes even if such money is not spent effectively and the invasions are not actually reduced.

## Conclusions

We have proposed GIRAE, a simple approach for calculating the per-unit effects of alien species, be they ecological impacts, damage costs, or management expenditure. This approach provides the means to disentangle the per-unit effect, range size and local abundance contribution to total impact for a wide range of taxonomic groups. We illustrated it for plant data using money spent on management, but other taxonomic groups and types of impact can be considered, using appropriate metrics for range size and local abundance. Two methods, the species-specific and the multi-species methods, can be used depending on the available data for each species. By disentangling the different components of impact, GIRAE paves the way towards further exploration of how characteristics of the alien species and recipient environments, and their interactions, influence these components. Such insights are crucial for designing and prioritising appropriate management actions, at both the regional and national scales, and moving towards objective frameworks for anticipating and responding to the impacts of alien species.

## Supplementary Information

Below is the link to the electronic supplementary material.

Supplementary file1 (DOCX 3856 kb)

## Data Availability

Data from van Wilgen et al. (2012) are directly accessible from the original article. The SAPIA database can be accessed by contacting the South Africa National Biodiversity Institute—SANBI (http://biodiversityadvisor.sanbi.org/contact/).
