# Supplementary material for: GIRAE: a generalised approach for linking the total impact of invasion to species' range, abundance and per-unit effects
Source: Biol Invasions. 2022 Jun 11;24(10):3147–67. doi: 10.1007/s10530-022-02836-0 (PMC9482606; doi:10.1007/s10530-022-02836-0)
Supplement: Supplementary file 1 — Supplementary file1 (DOCX 3856 kb) [file 10530_2022_2836_MOESM1_ESM.docx]

Supporting information

For " G-IRAE: a Generalised approach for linking the total Impact of invasion to species' Range, Abundance and per-unit Effects", by Guillaume Latombe, Jane A. Catford, Franz Essl, Bernd Lenzner, David M. Richardson, John R. U. Wilson & Melodie A. McGeoch.

This document contains additional figures presenting results from analyses.


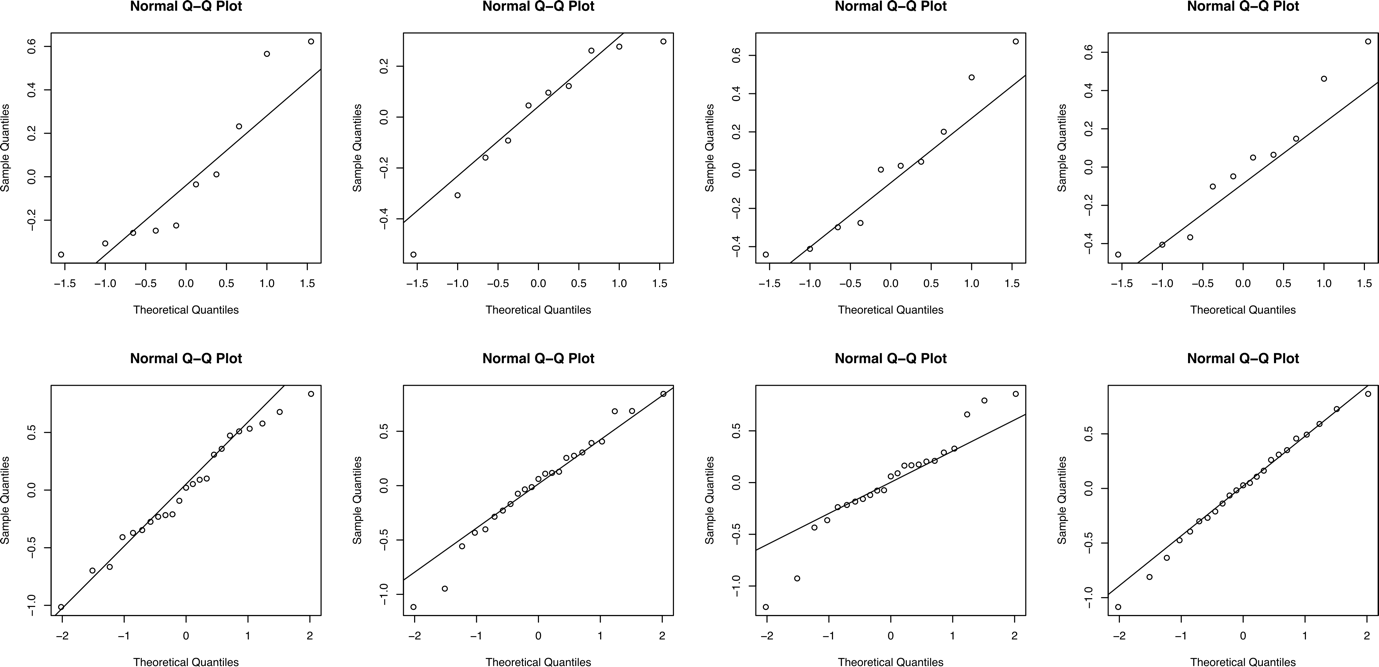


**Figure S1.** Q-Q plots of model residuals for four random replicates out of 1000 for the species-specific approach for models on acacia data (first row) and models on other species (second row), calculated after randomly sampling SAPIA records.


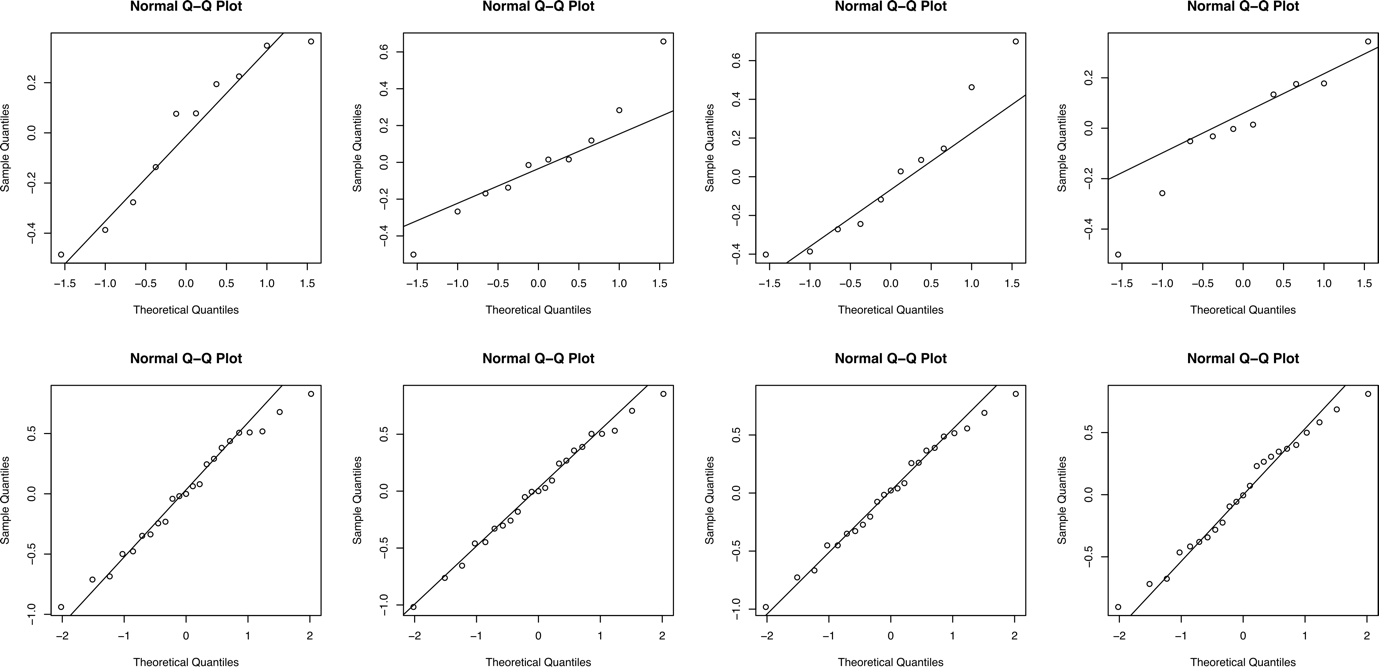


**Figure S2.** Q-Q plots of model residuals for four random replicates out of 1000 for the species-specific approach for models on acacia data (first row) and models on other species (second row), calculated after sampling predominantly SAPIA records with high abundance.


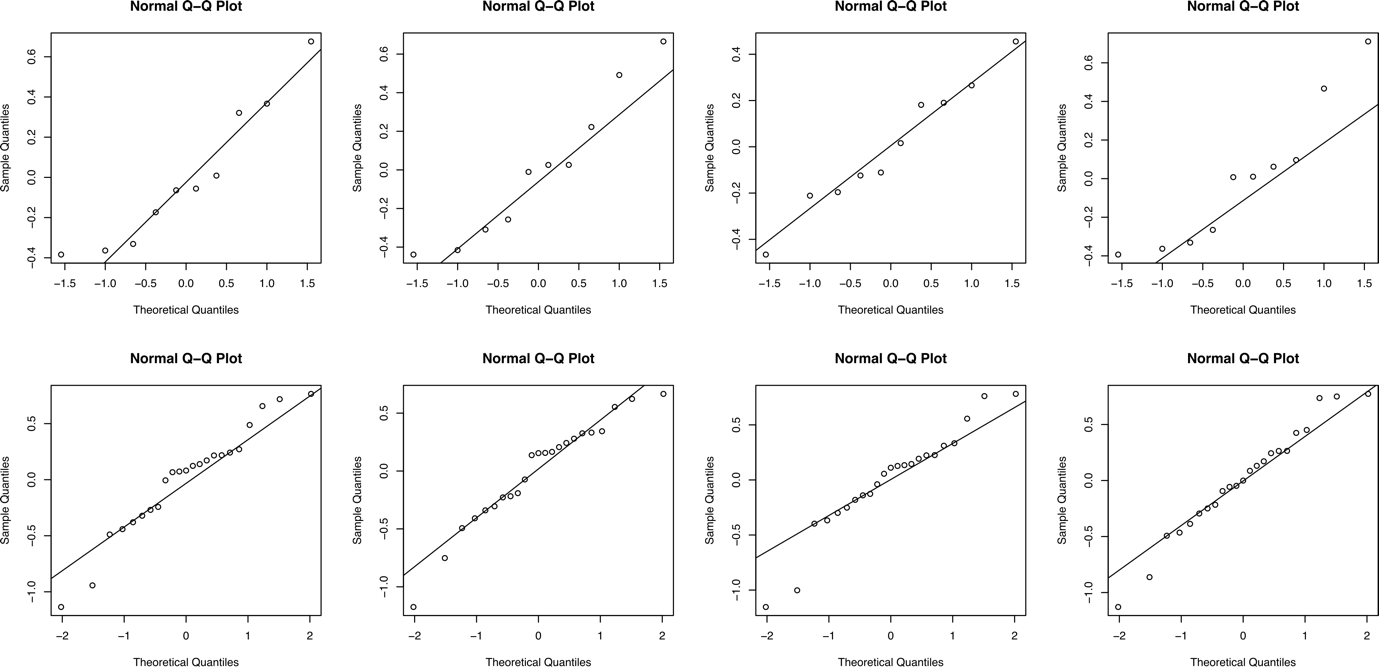


**Figure S3.** Q-Q plots of model residuals for four random replicates out of 1000 for the species-specific approach for models on acacia data (first row) and models on other species (second row), calculated after sampling predominantly SAPIA records with low abundance.


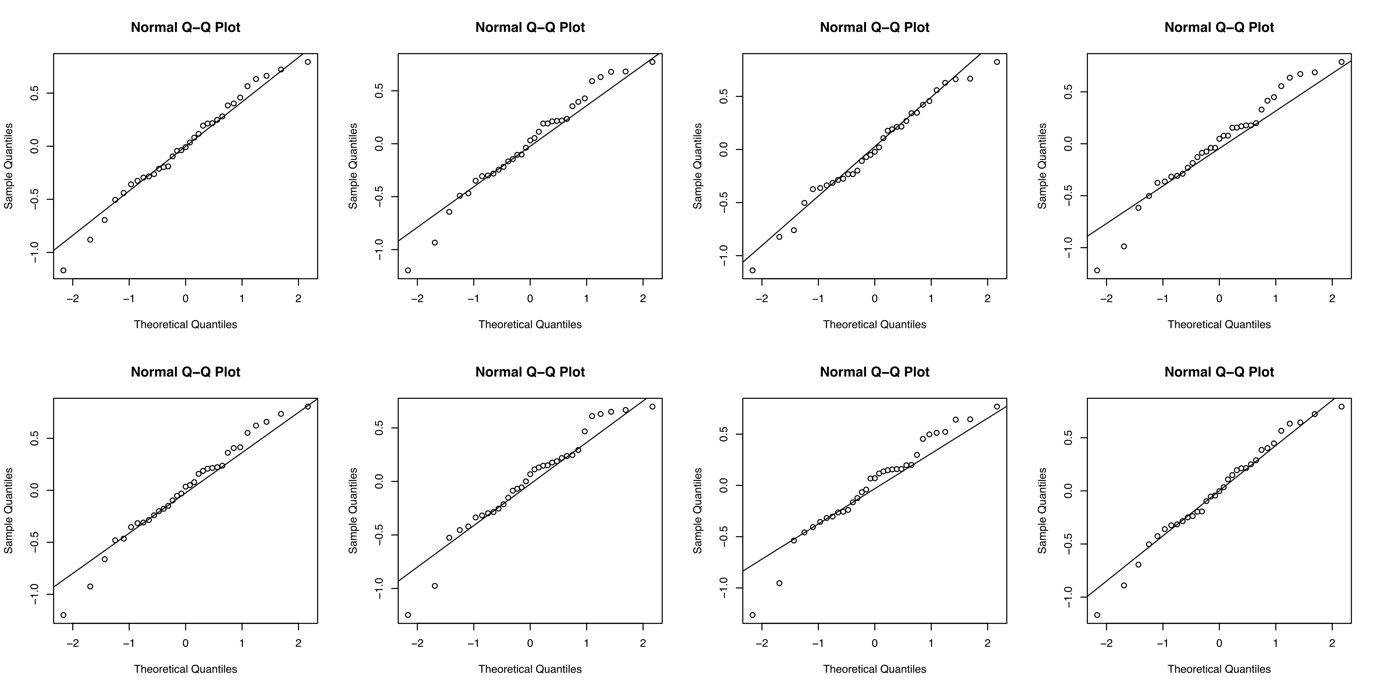


**Figure S4.** Q-Q plots of model residuals for eight random replicates out of 1000, calculated after randomly sampling SAPIA records.


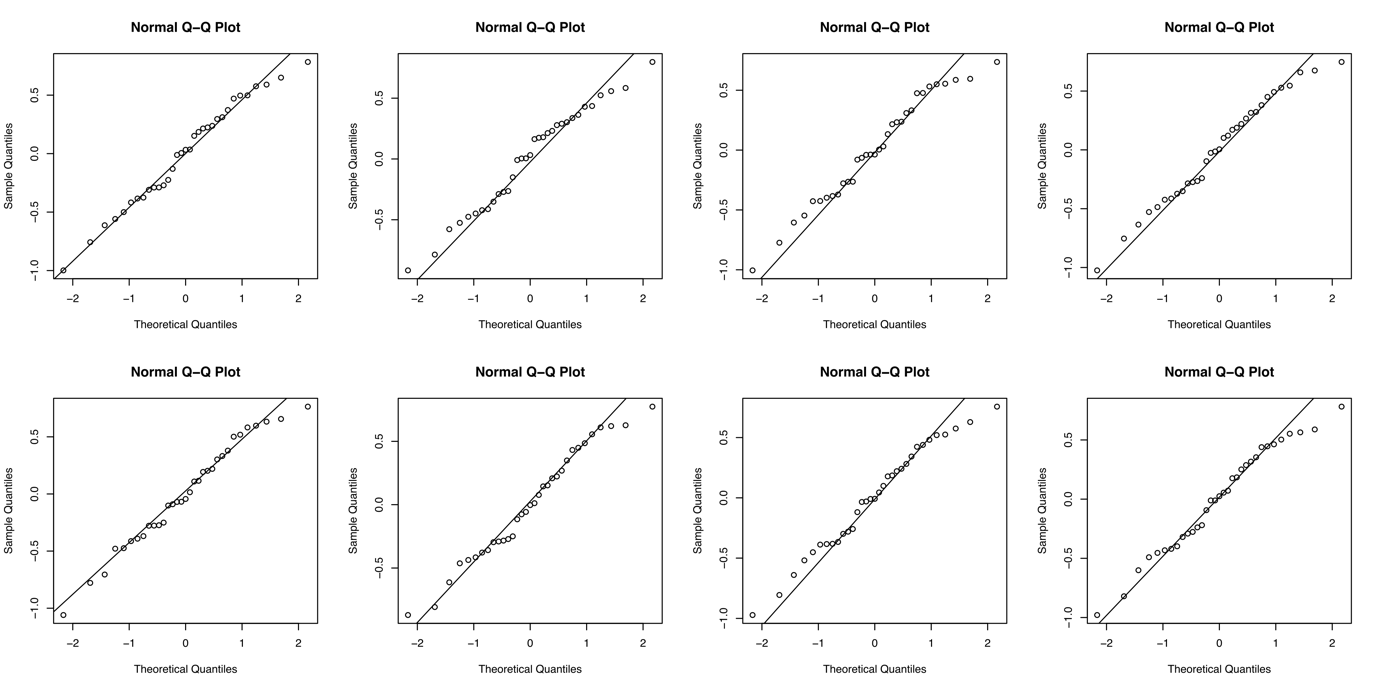


**Figure S5.** Q-Q plots of model residuals for eight random replicates out of 1000, calculated after sampling predominantly SAPIA records with high abundance.


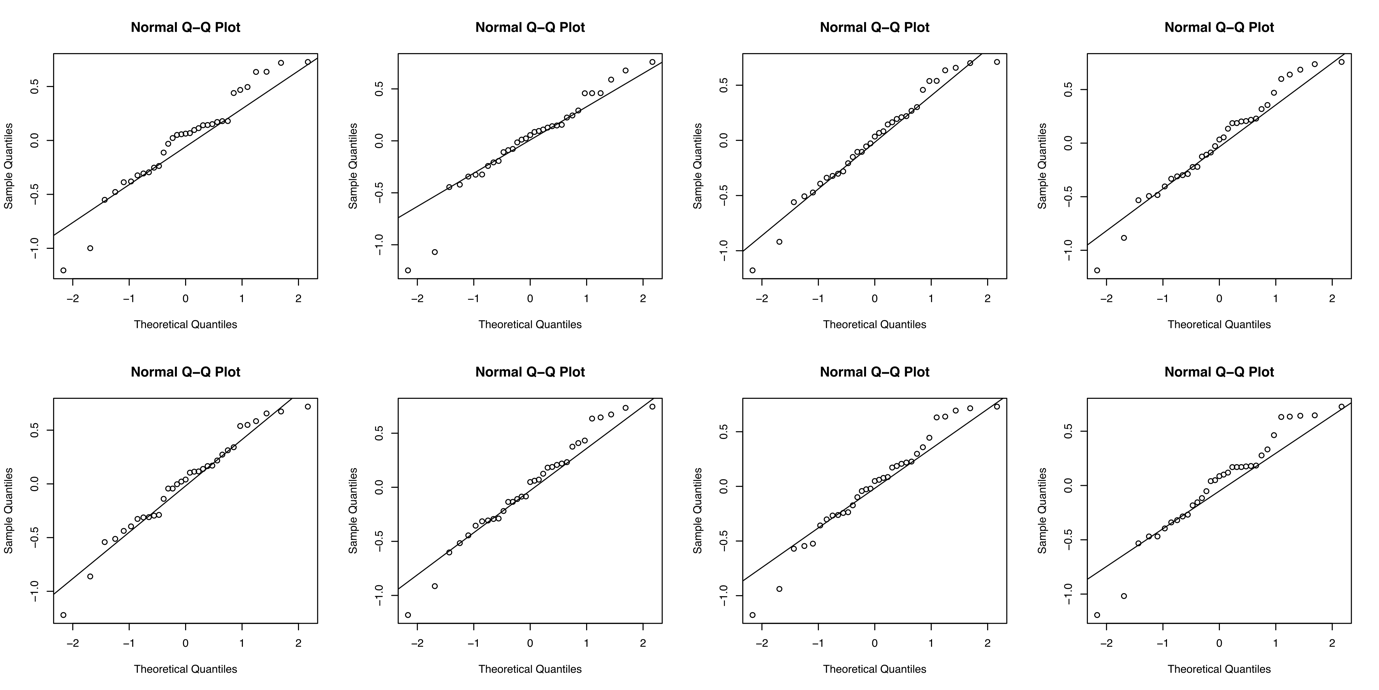


**Figure S6.** Q-Q plots of model residuals for eight random replicates out of 1000, calculated after sampling predominantly SAPIA records with low abundance.


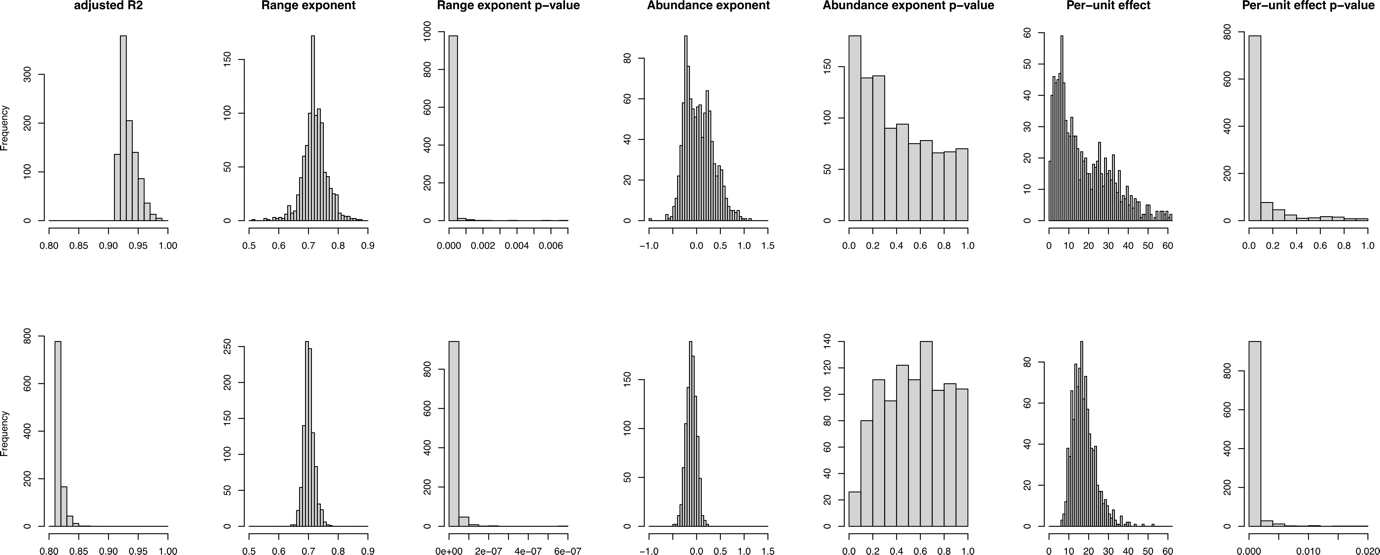


**Figure S7.** Model results calculated after randomly sampling SAPIA records for the species-specific approach. a) Results for acacias. b) Results for all other species combined.


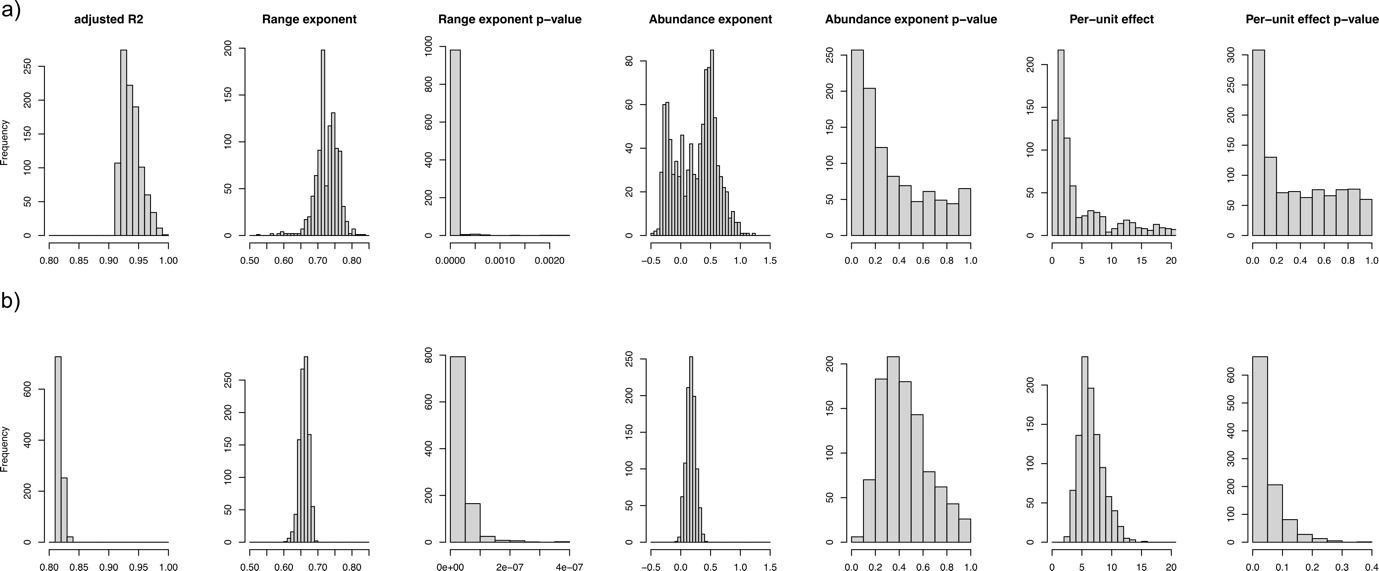


**Figure S8.** Model results calculated after sampling predominantly SAPIA records with high abundance for the species-specific approach. a) Results for acacias. b) Results for all other species combined.


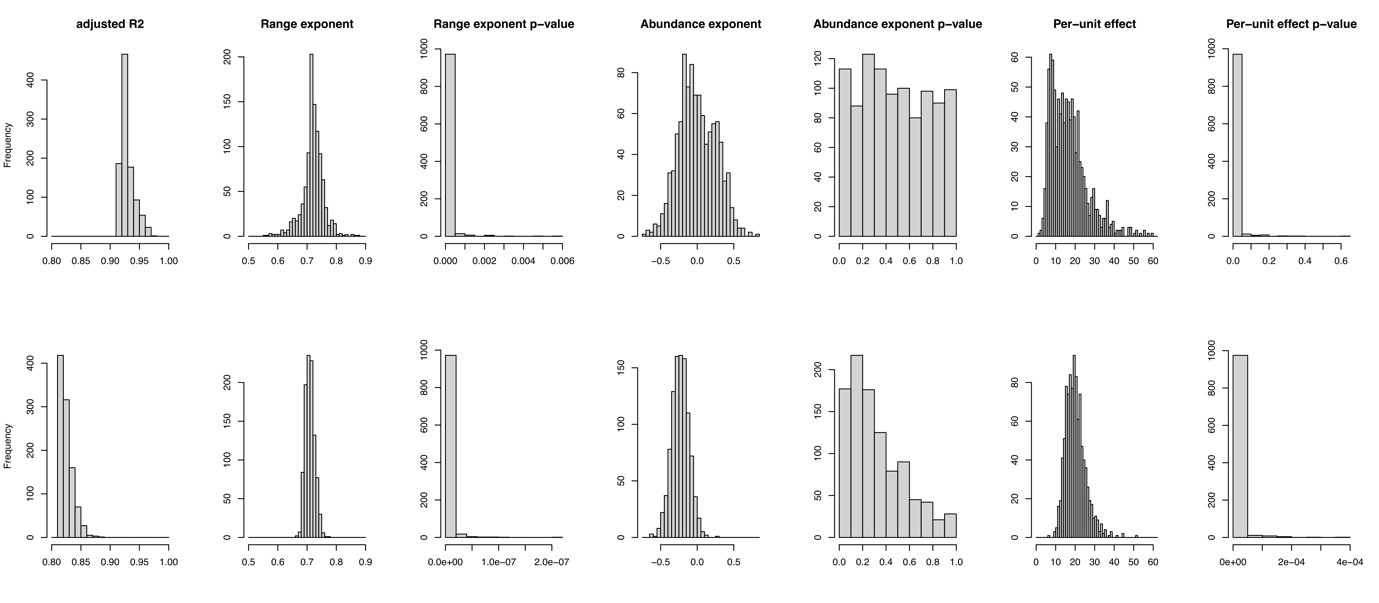


**Figure S9.** Model results calculated after sampling predominantly SAPIA records with low abundance for the species-specific approach. a) Results for acacias. b) Results for all other species combined.


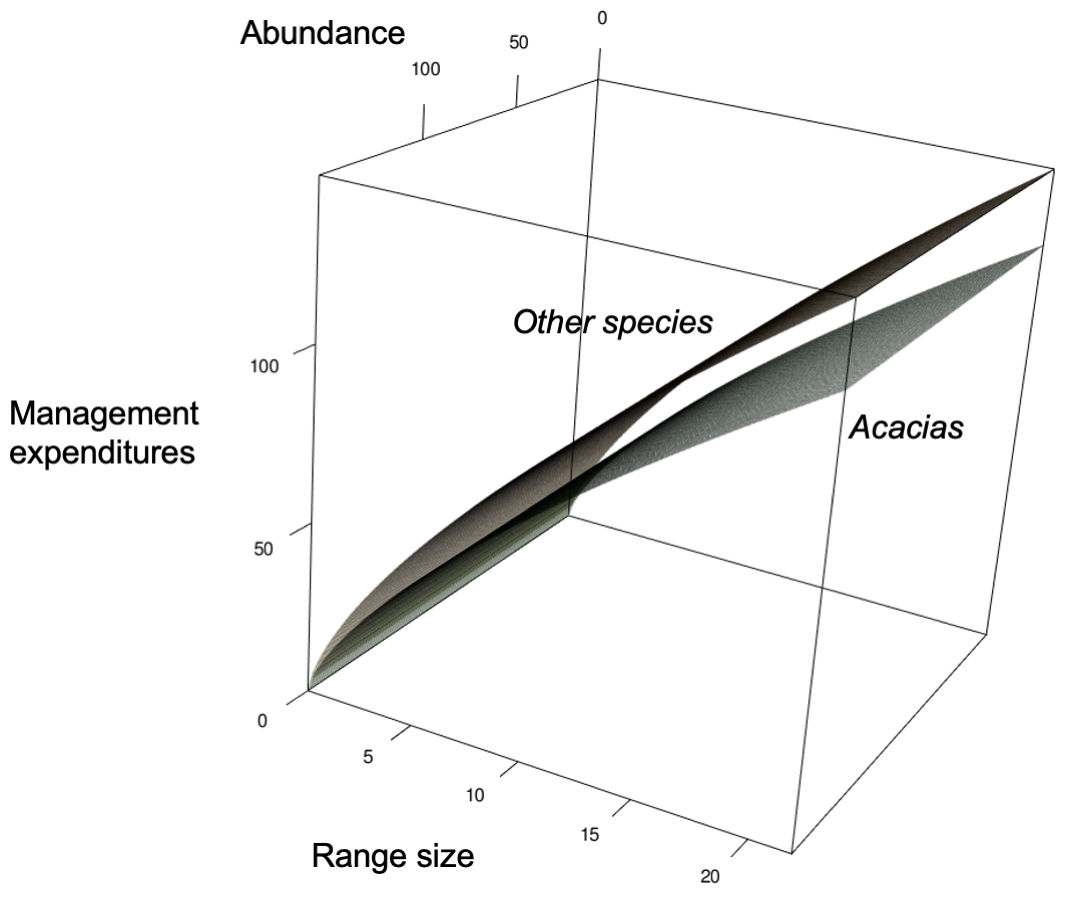


**Figure S10.** Relationship between management expenditures, range size and local abundance, after fitting Equation 6 for Acacias and other species and randomly sampling SAPIA records.


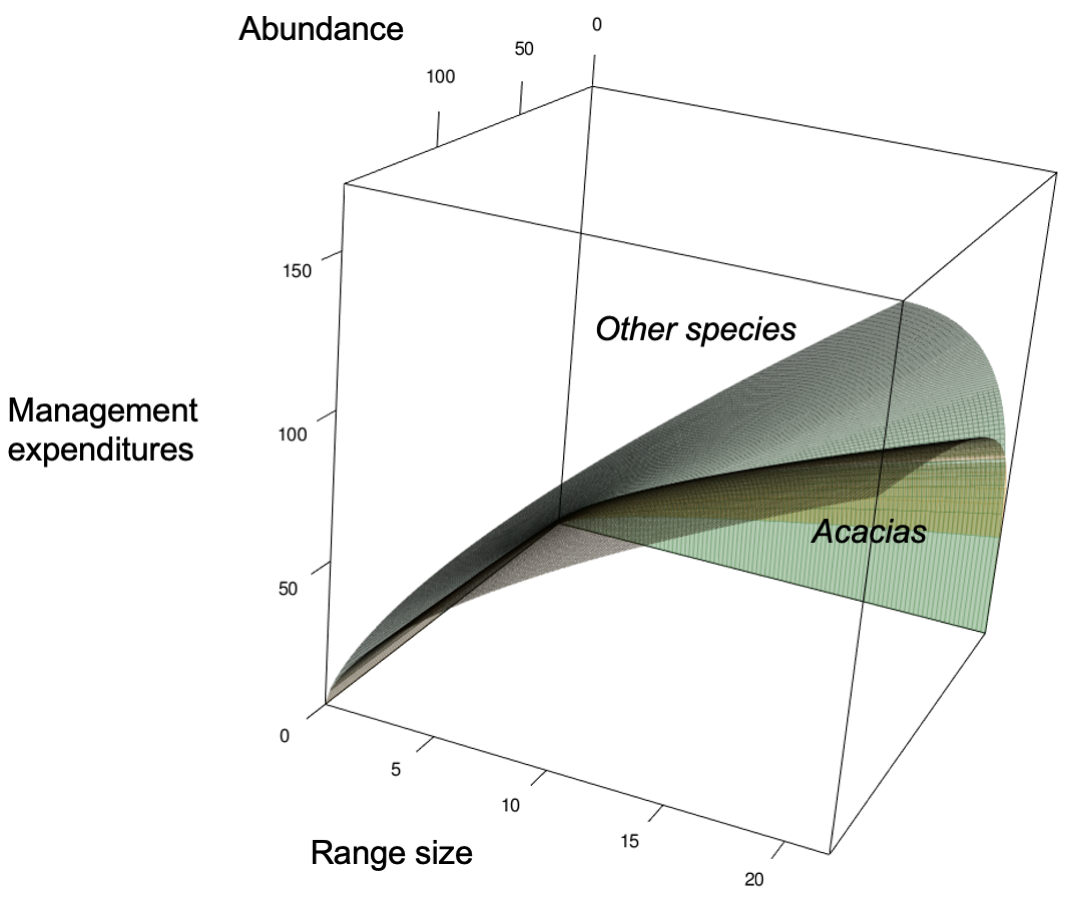


**Figure S11.** Relationship between management expenditures, range size and local abundance, after fitting Equation 6 for Acacias and other species and sampling predominantly SAPIA records with high abundance


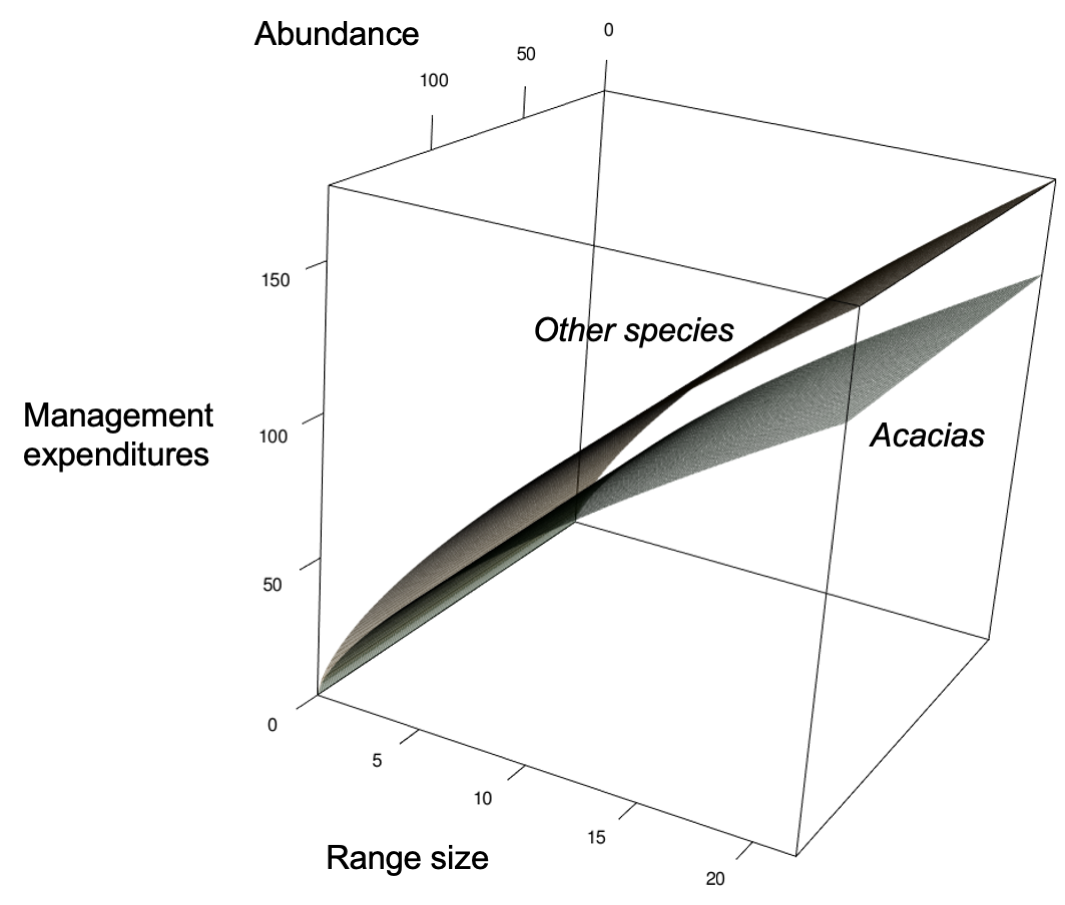


**Figure S12.** Relationship between management expenditures, range size and local abundance, after fitting Equation 6 for Acacias and other species and sampling predominantly SAPIA records with low abundance.


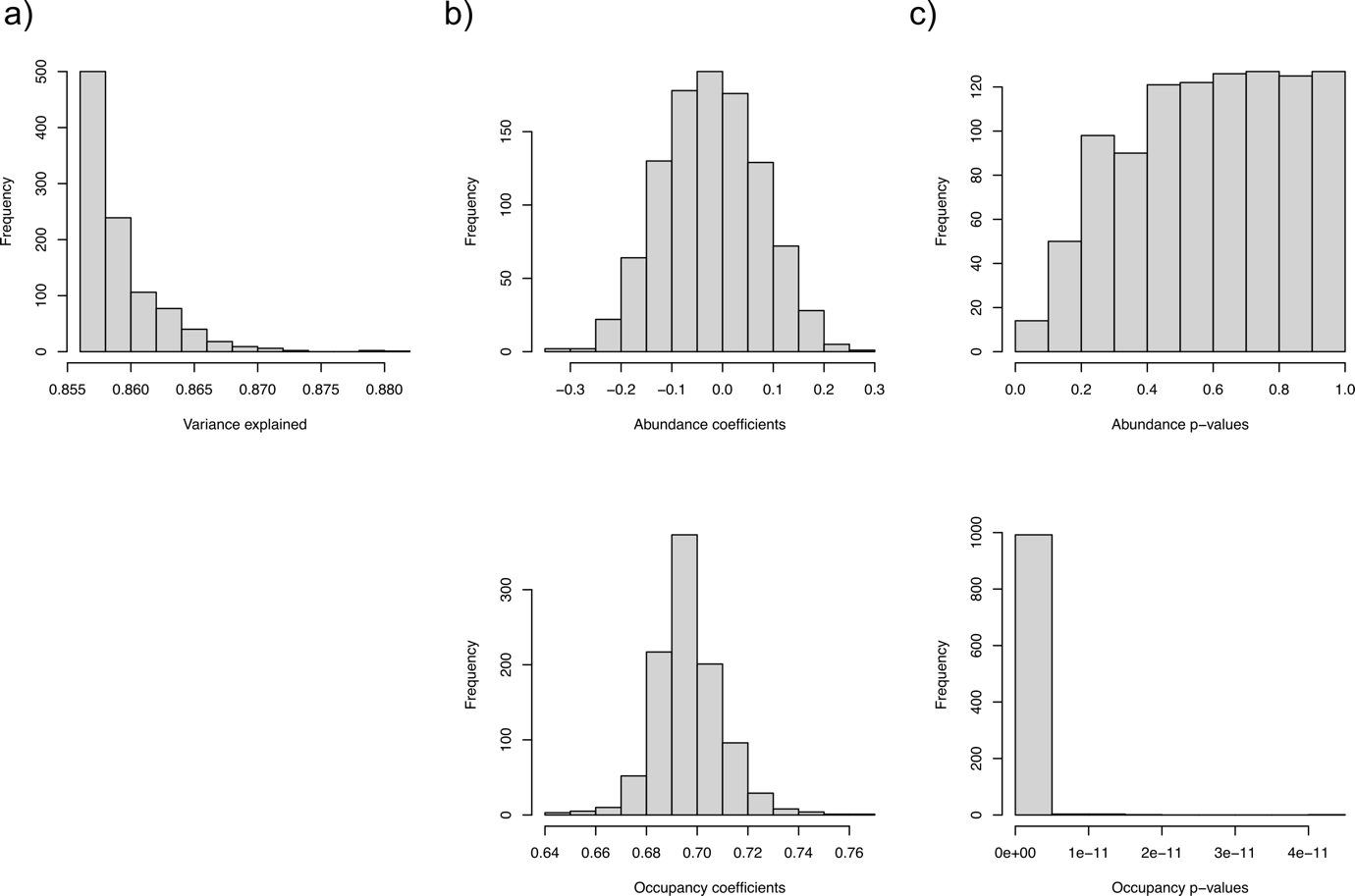


**Figure S13.** Model results calculated after randomly sampling SAPIA records. a) Distribution of the variance explained by the models for the 1000 replicates. b) Distributions of the α and β coefficients for abundance and occupancy. c) Distributions of the p-values of the α and β coefficients.

**
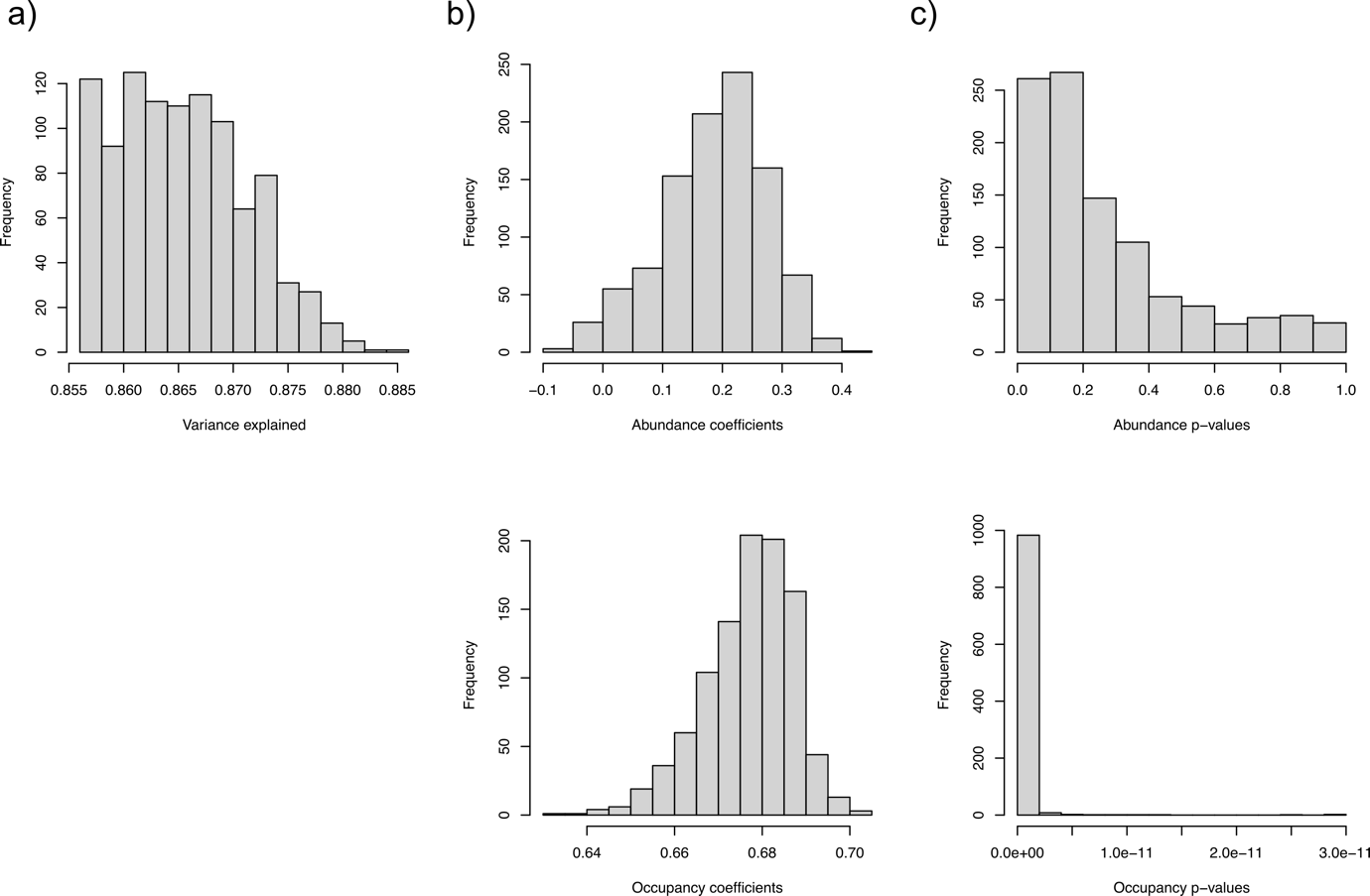
**

**Figure S14.** Model results calculated after sampling predominantly SAPIA records with high abundance. a) Distribution of the variance explained by the models for the 1000 replicates. b) Distributions of the α and β coefficients for abundance and occupancy. c) Distributions of the p-values of the α and β coefficients.


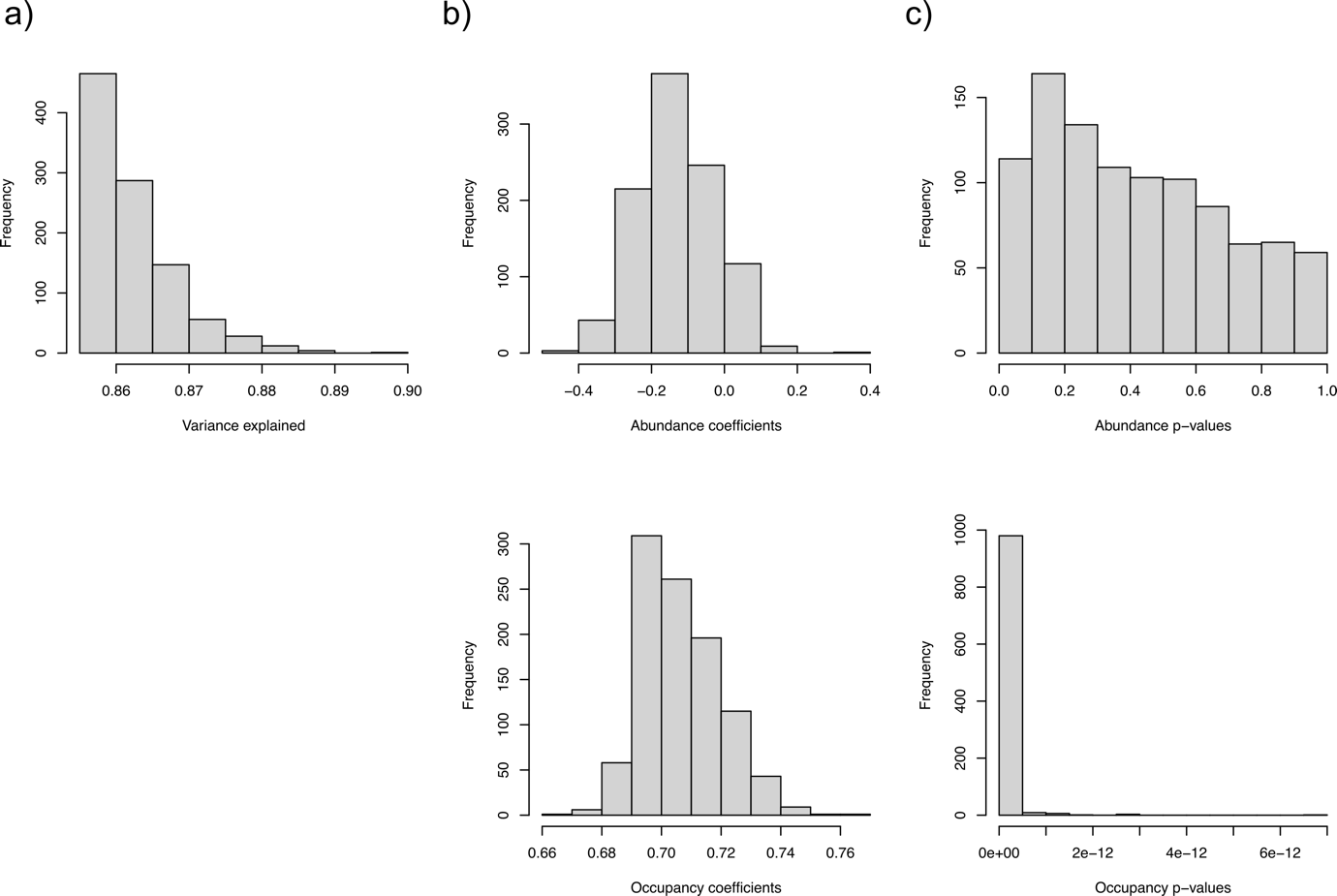


**Figure S15.** Model calculated after sampling predominantly SAPIA records with low abundance. a) Distribution of the variance explained by the models for the 1000 replicates. b) Distributions of the α and β coefficients for abundance and occupancy. c) Distributions of the p-values of the α and β coefficients.


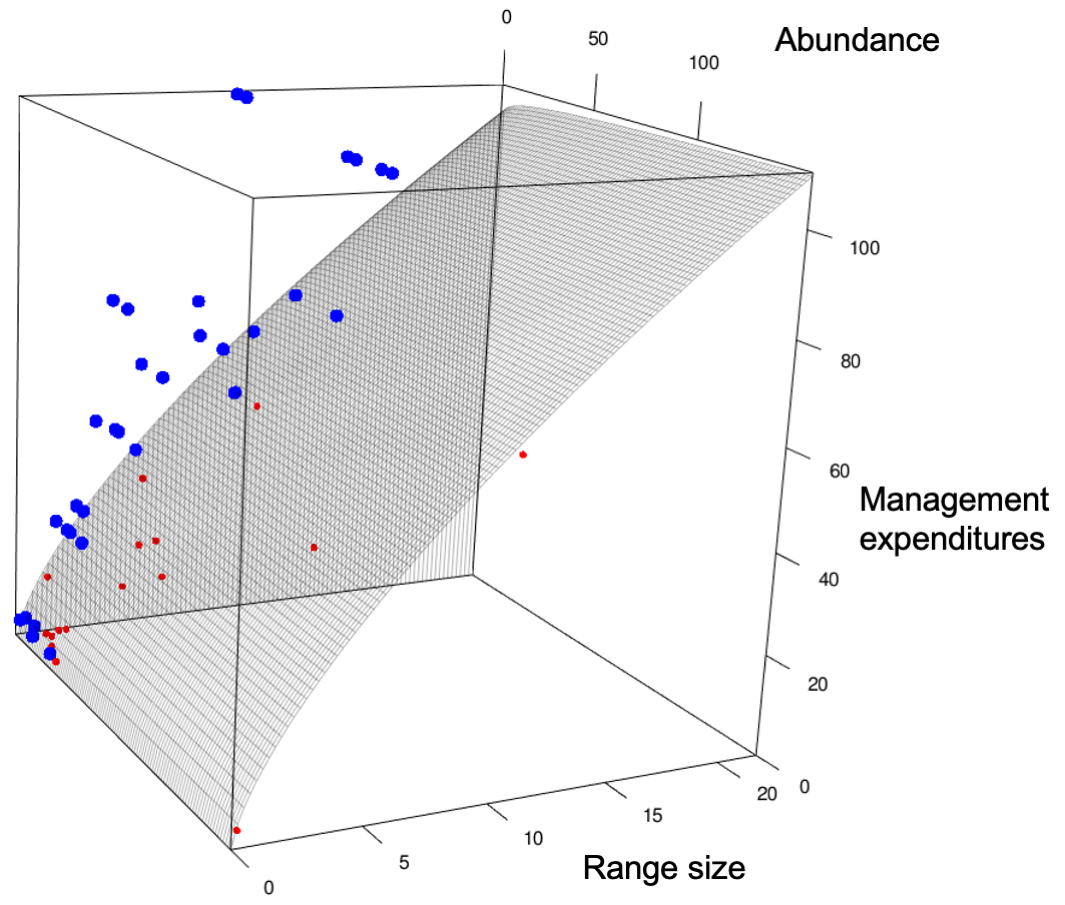


**Figure S16.** Relationship between management expenditures, range size and local abundance, after fitting Equation 8 and randomly sampling SAPIA records. The big blue points represent the management expenditures for species that are underestimated by the relationship (i.e. their per-unit management expenditures are above the baseline value), and the small red points represent the management expenditures for species that are overestimated by the relationship.


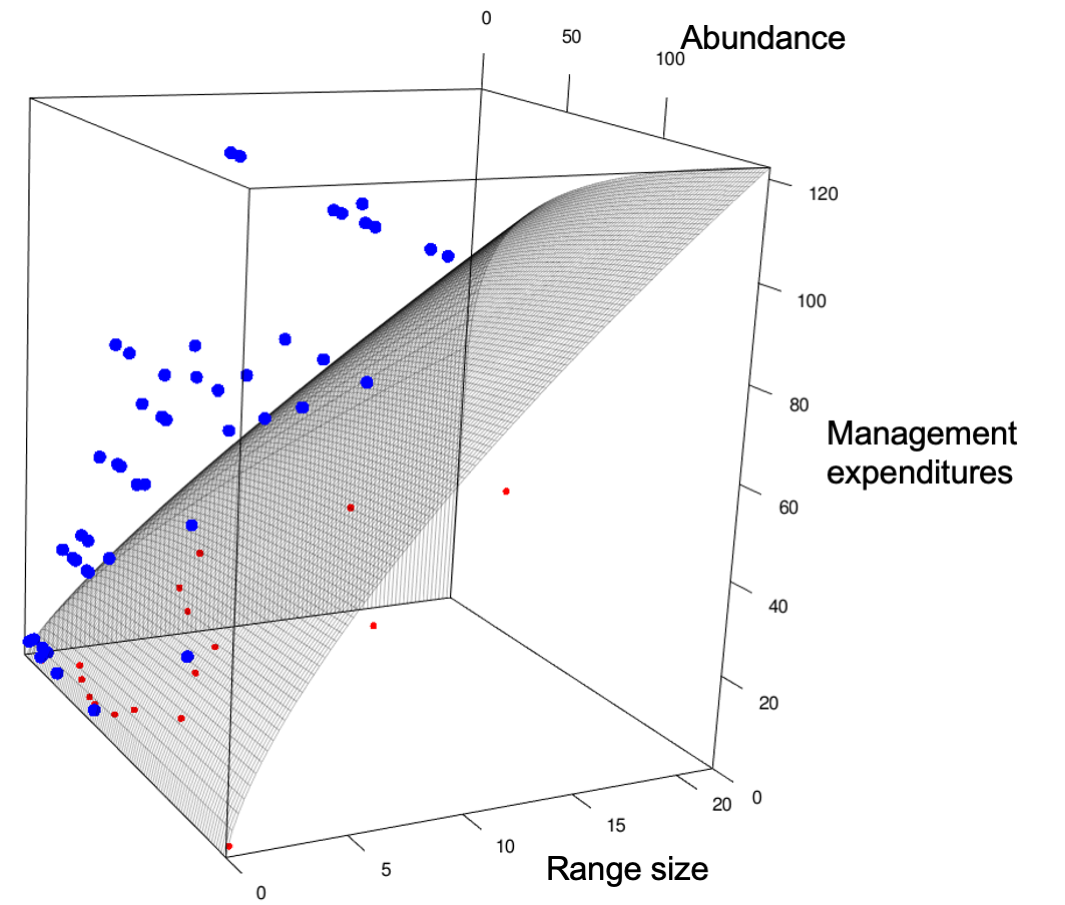


**Figure S17.** Relationship between management expenditures, range size and local abundance, after fitting Equation 8 and sampling predominantly SAPIA records with high abundance. The big blue points represent the management expenditures for species that are underestimated by the relationship (i.e. their per-unit management expenditures are above the baseline value), and the small red points represent the management expenditures for species that are overestimated by the relationship.


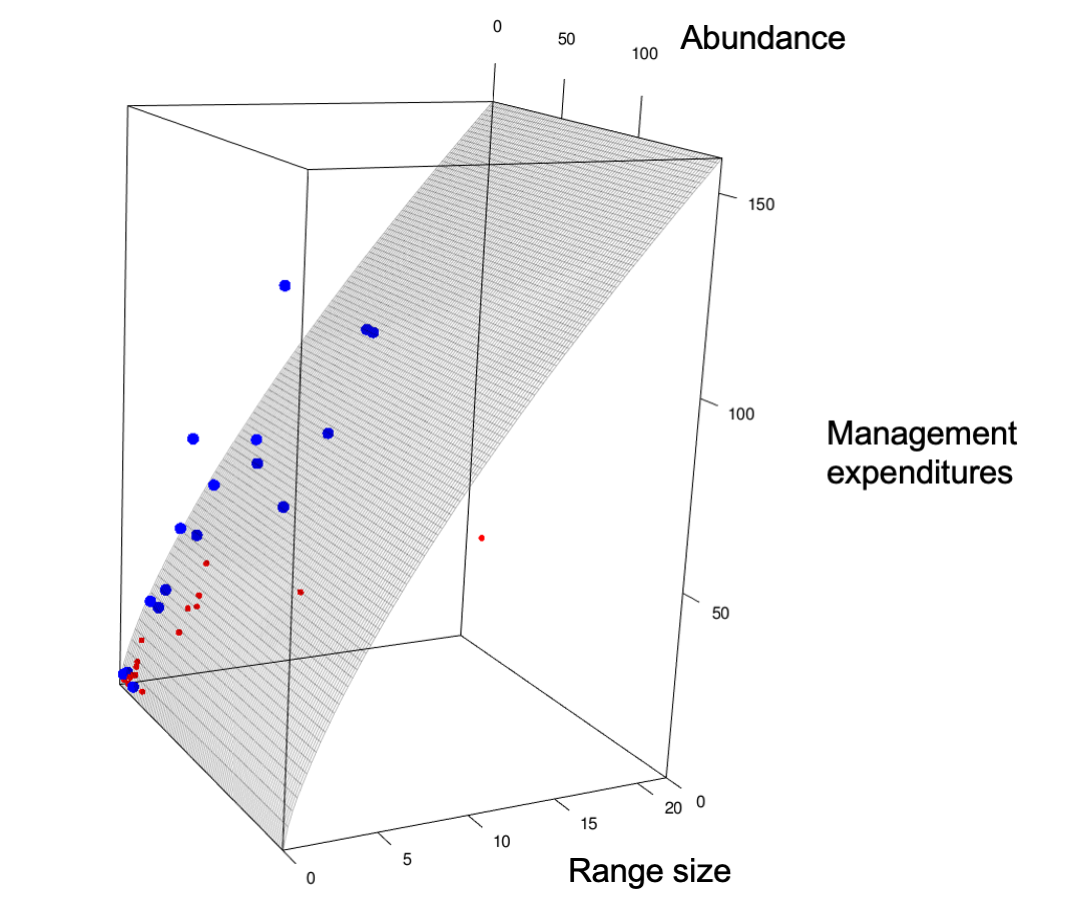


**Figure S18.** Relationship between management expenditures, range size and local abundance, after fitting Equation 8 and sampling predominantly SAPIA records with low abundance. The big blue points represent the management expenditures for species that are underestimated by the relationship (i.e. their per-unit management expenditures are above the baseline value), and the small red points represent the management expenditures for species that are overestimated by the relationship.


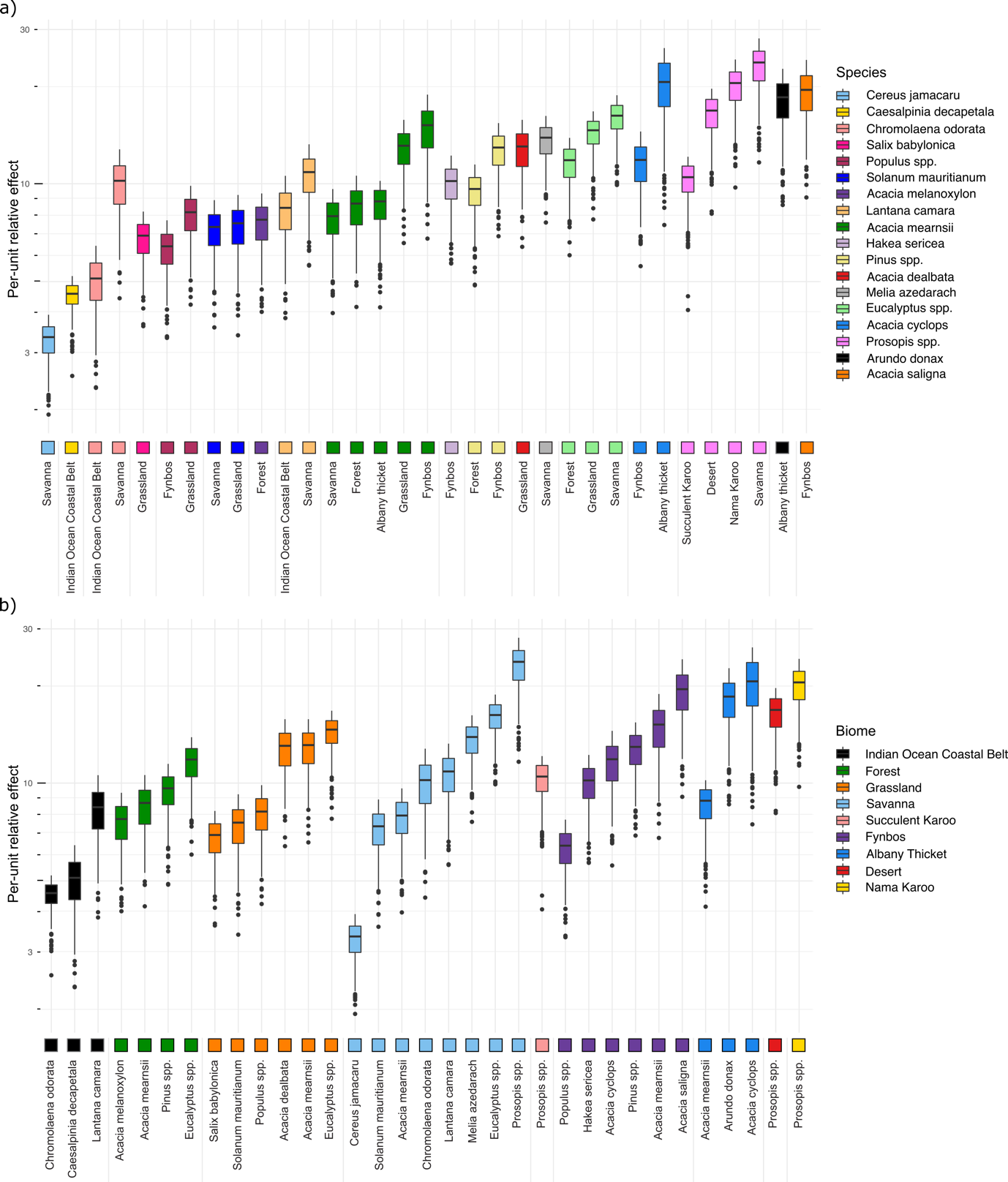


**Figure S19.** Distributions of per-unit relative effects (here per-unit relative management costs, i.e. money spent per-unit on management) of alien species managed by the Working for Water program in South Africa between 1999 and 2008, in the different biomes of South Africa, over the 1000 replicates, calculated after randomly sampling records. Per-unit cost values should be interpreted in a relative rather than absolute fashion, due to the lack of absolute meaning for the abundance values. a) Species are distinguished by different colours and ordered by their median per-unit cost over all biomes where they were managed. b) Biomes are distinguished by different colours and ordered by their median per-unit cost over all managed species they contain.


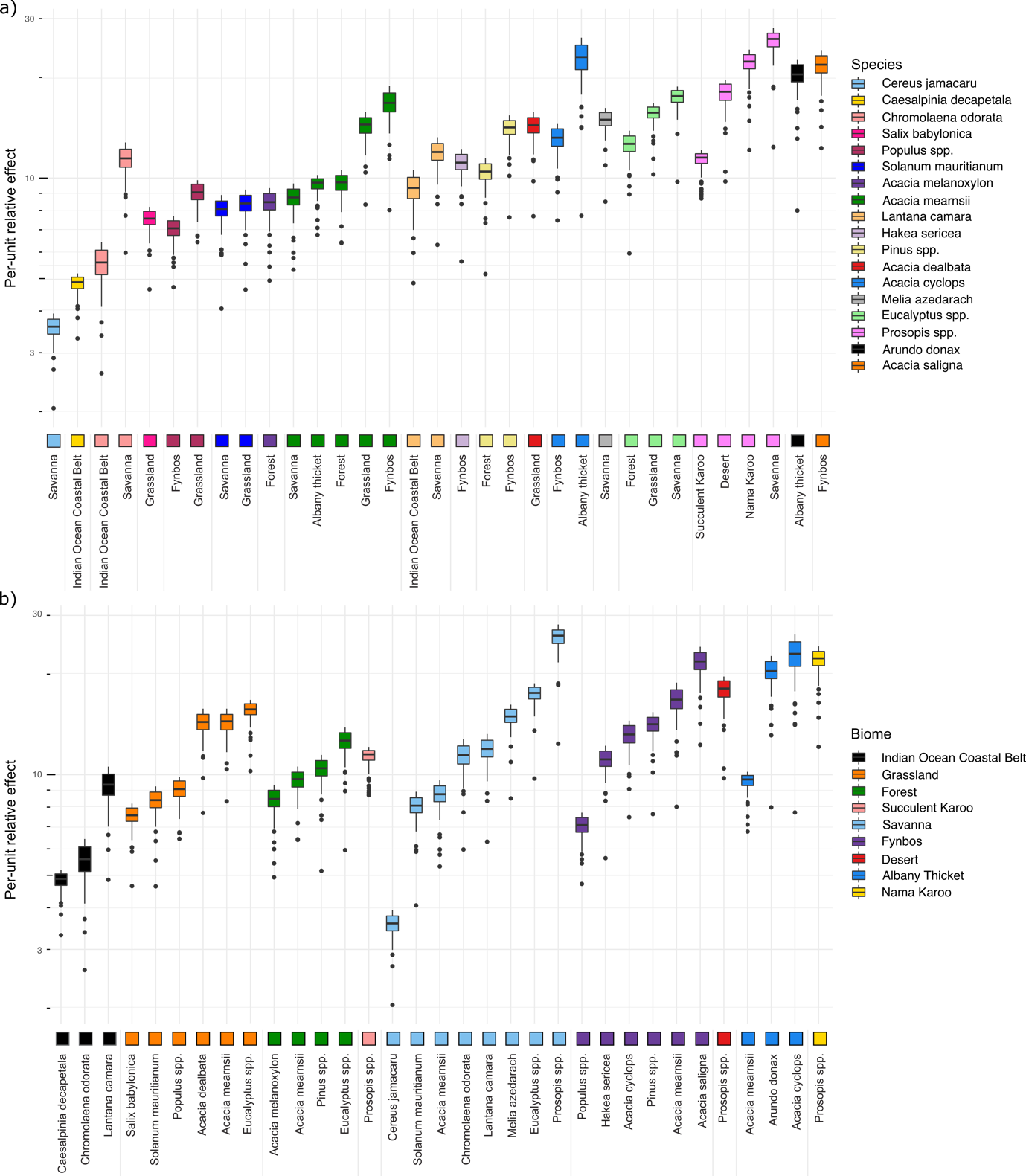


**Figure S20.** Distributions of per-unit relative effects (here per-unit relative management costs, i.e. money spent per-unit on management) of alien species managed by the Working for Water program in South Africa between 1999 and 2008, in the different biomes of South Africa, over the 1000 replicates, calculated after sampling predominantly records with low abundance. Per-unit cost values should be interpreted in a relative rather than absolute fashion, due to the lack of absolute meaning for the abundance values. a) Species are distinguished by different colours and ordered by their median per-unit cost over all biomes where they were managed. b) Biomes are distinguished by different colours and ordered by their median per-unit cost over all managed species they contain.
